# Esrrb Complementation Rescues Development of *Nanog*-Null Germ Cells

**DOI:** 10.1016/j.celrep.2017.12.060

**Published:** 2018-01-09

**Authors:** Man Zhang, Harry G. Leitch, Walfred W.C. Tang, Nicola Festuccia, Elisa Hall-Ponsele, Jennifer Nichols, M. Azim Surani, Austin Smith, Ian Chambers

**Affiliations:** 1 MRC Centre for Regenerative Medicine, Institute for Stem Cell Research, School of Biological Sciences, University of Edinburgh, 5 Little France Drive, Edinburgh EH16 4UU, Scotland; 2 Wellcome Trust-Medical Research Council Stem Cell Institute, University of Cambridge, Cambridge CB2 1QR, United Kingdom; 3 Wellcome Trust/Cancer Research UK Gurdon Institute, Tennis Court Road, University of Cambridge, Cambridge CB2 1QN, United Kingdom; 4 Department of Physiology, Development and Neuroscience, Downing Street, University of Cambridge, Cambridge CB2 3EG, United Kingdom; 5 Department of Biochemistry, University of Cambridge, Tennis Court Road, Cambridge CB2 1GA, United Kingdom

**Keywords:** primordial germ cells, naive pluripotency, competence, transcription factors, PGCLCs

## Abstract

The transcription factors (TFs) Nanog and Esrrb play important roles in embryonic stem cells (ESCs) and during primordial germ-cell (PGC) development. *Esrrb* is a positively regulated direct target of NANOG in ESCs that can substitute qualitatively for *Nanog* function in ESCs. Whether this functional substitution extends to the germline is unknown. Here, we show that germline deletion of *Nanog* reduces PGC numbers 5-fold at midgestation. Despite this quantitative depletion, *Nanog*-null PGCs can complete germline development in contrast to previous findings. PGC-like cell (PGCLC) differentiation of *Nanog*-null ESCs is also impaired, with *Nanog*-null PGCLCs showing decreased proliferation and increased apoptosis. However, induced expression of Esrrb restores PGCLC numbers as efficiently as Nanog. These effects are recapitulated *in vivo*: knockin of Esrrb to *Nanog* restores PGC numbers to wild-type levels and results in fertile adult mice. These findings demonstrate that Esrrb can replace Nanog function in germ cells.

## Highlights

• Germline deletion of *Nanog* reduces PGC numbers but does not abolish PGC development
• Without Nanog, PGCLCs form ineffectively with less proliferation and more apoptosis
• The Nanog target gene Esrrb can rescue PGCLC differentiation of *Nanog*^−/−^ ESCs
• Knockin of *Esrrb* at the *Nanog* locus restores PGC development efficiency

## Graphical Abstract

**Figure:**
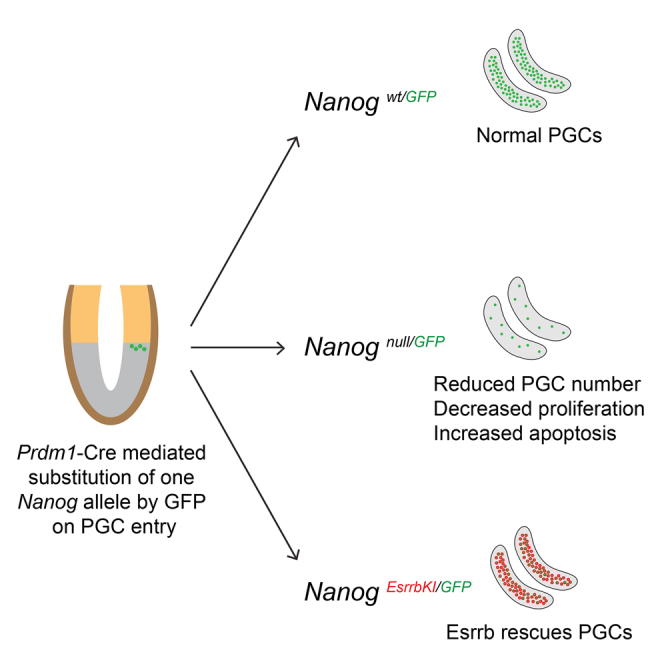

## Teaser

Although transcription factors functional in naive pluripotent cells are also expressed in primordial germ cells (PGCs), their PGC role remains unclear. Here, Zhang et al. show that, without *Nanog*, PGCs form ineffectively but that normal PGC development can be restored by induced expression of the NANOG target gene *Esrrb*.

## Introduction

Naive pluripotency is established in epiblast cells of the mouse blastocyst (Boroviak et al., 2014, Brook and Gardner, 1997). The transcription factors (TFs) *Oct4*, *Sox2*, and *Nanog* are required to establish epiblast identity and are fundamental pluripotency regulators *in vivo* and *in vitro* (Festuccia et al., 2013). Following implantation, the epiblast enters a transitional phase in which cells remain uncommitted and functionally pluripotent (Beddington, 1982, Osorno et al., 2012, Tam and Zhou, 1996). At this point, expression of OCT4 and SOX2, but not NANOG or other naive TFs, is maintained (Smith, 2017). Mouse primordial germ cells (PGCs) are induced from the pluripotent post-implantation epiblast early on embryonic day (E)6 (Ohinata et al., 2005) and upregulate expression of many naive pluripotency genes following specification (Kurimoto et al., 2008). PGCs do not contribute to chimeras when injected into blastocysts (Leitch et al., 2014), but possess a latent capacity to reacquire pluripotency, which can be revealed *in vivo* during teratocarcinogenesis (Stevens, 1983) or by the derivation *in vitro* of naive pluripotent stem cell lines called embryonic germ cells (Leitch et al., 2013, Matsui et al., 1992, Resnick et al., 1992). Furthermore, PGC development is dependent on the expression of pluripotency TFs. Conditional deletion of either *Oct4* or *Sox2* results in PGC death (Campolo et al., 2013, Kehler et al., 2004). *Nanog*-null embryonic stem cells (ESCs) exhibit broad differentiation potential, including to migratory PGCs, but contribution to germ cells at E12.5 was not observed in our previous study (Chambers et al., 2007). Induced knockdown of *Nanog* in PGCs results in significant alteration of their transcriptional program and subsequent apoptosis (Yamaguchi et al., 2009). Induction of PGC-like cells (PGCLCs) *in vitro* is impaired in the absence of *Nanog*, whereas exogenous *Nanog* improves PGCLC yield (Murakami et al., 2016), in keeping with *in vivo* findings.

*Nanog* is essential for the specification of pluripotency *in vivo* (Mitsui et al., 2003, Silva et al., 2009). However, *Nanog*-null ESCs can be maintained, albeit with a reduced self-renewal efficiency (Chambers et al., 2007). The orphan nuclear receptor Esrrb is a regulator of ESC self-renewal (Festuccia et al., 2012, Ivanova et al., 2006, Martello et al., 2012) and influences PGC numbers *in vivo* (Mitsunaga et al., 2004). *Esrrb* is also a direct NANOG target (Festuccia et al., 2012). Deletion of *Esrrb* abolishes the ability of NANOG to confer leukemia inhibitory factor (LIF) independence in ESCs (Festuccia et al., 2012). Furthermore, ESRRB can compensate for NANOG function in epiblast stem cell (EpiSC) reprogramming and in induced pluripotent stem cell (iPSC) generation (Festuccia et al., 2012). Thus, Esrrb is a key downstream mediator of Nanog function in the maintenance and establishment of pluripotency *in vitro*. Here, we reassess the requirement for Nanog in PGCs and investigate whether ESRRB can compensate for NANOG function during PGC development.

## Results

### Conditional Deletion of Nanog Reduces PGC Numbers

**Figure 1:**
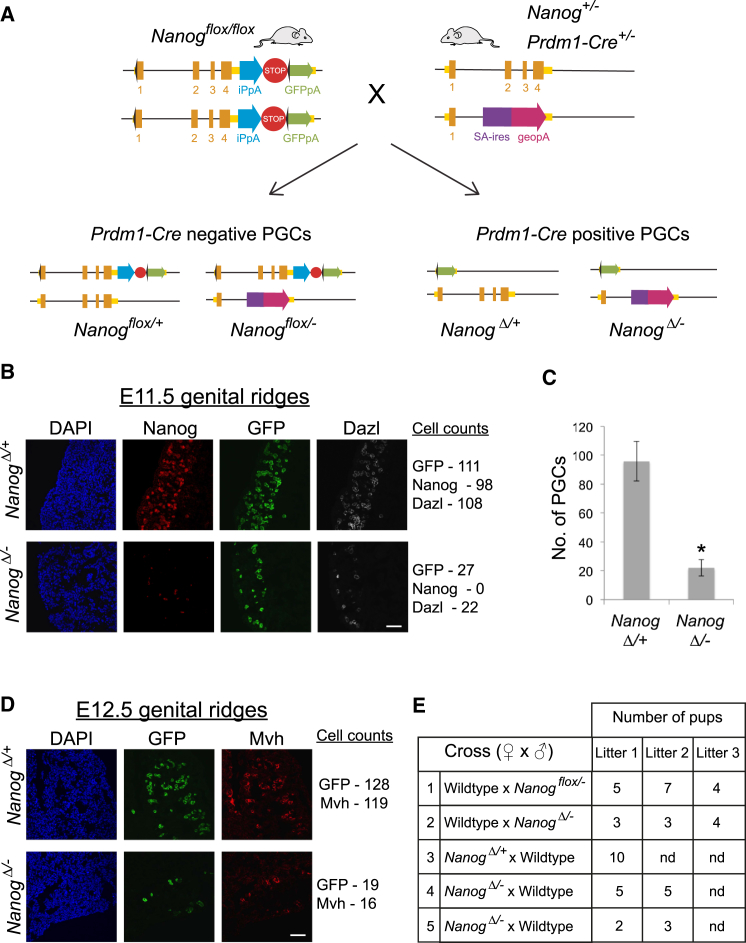
Conditional Deletion of *Nanog* Reduces PGC Numbers (A) Strategy for *Nanog* conditional knockout. *Nanog*^*flox/flox*^ females are crossed with *Nanog*^*+/−*^*; Prdm1-Cre* male mice. As *Prdm1-Cre* is heterozygous, one in four embryos will have germline deletion of *Nanog* (*Nanog*^*Δ/−*^). (B) E11.5 genital ridge sections from *Nanog*^*Δ/−*^ and control embryos immunostained for Nanog, Dazl, and GFP and counterstained with 4',6-diamidino-2-phenylindole (DAPI) (scale bar, 50 μm). (C) Cell counts of PGCs in *Nanog*^*Δ/−*^ and control genital ridges at E11.5. PGCs identified by co-staining for Oct4 and either Dazl or Mvh. The mean (± SD) of two biological and technical replicates for each sample are shown. ^∗^p < 0.05 (unpaired Student’s t test). (D) E12.5 genital ridges from *Nanog*^*Δ/−*^ and control embryos immunostained for GFP and Mvh and counterstained with DAPI (scale bar, 50 μm). (E) Table of breeding data for adult *Nanog*^*Δ/−*^ mice. Both male (row 2) and female (row 4 and 5) *Nanog*^*Δ/−*^ mice are fertile. See also Figure S1.

To assess whether Nanog is required cell autonomously in PGCs, a conditional knockout strategy was used. Mice homozygous for a *Nanog* conditional allele (*Nanog*^*flox/flox*^) (Chambers et al., 2007) were crossed with *Nanog* heterozygous mice (*Nanog*^*+/−*^) (Mitsui et al., 2003) harboring the *Prdm1-Cre-BAC* transgene (Ohinata et al., 2005) (Figure 1A). One in four offspring carried the *Nanog* null (−) and conditionally deleted (Δ) alleles in PGCs. As Prdm1-Cre-mediated excision has been reported to be incomplete until after E10.5 (Campolo et al., 2013, Kim et al., 2014), genital ridges in control and mutant embryos were dissected at E11.5 and analyzed by immunofluorescence. NANOG protein was not detected in mutant genital ridges (Figure 1B). However, GFP-positive cells were present, indicating successful deletion of *Nanog* (Figure 1B). GFP-positive cells were positive for DAZL, indicating that these represent *Nanog*-null PGCs (Figure 1B). Compared with littermate controls, PGC numbers in *Nanog* mutant embryos were reduced 80% (Figure 1B and 1C). Surprisingly, a small number of GFP-positive mutant PGCs expressing MVH were also detected at E12.5 (Figure 1D). To establish whether these surviving *Nanog* mutant PGCs were developmentally competent, subsequent litters were allowed to go to term, and adult mutant mice of both sexes were test-crossed (Figures S1A and S1B). Male and female mutant mice were fertile (Figure 1E), passing either the knockout or conditionally deleted allele to their offspring (Figure S1C). These findings indicate that Prdm1-Cre-mediated deletion of *Nanog* reduces the PGC number, but suggest that *Nanog* might not be strictly required for germline development.

### Nanog Is Not Essential for Germline Development

**Figure 2:**
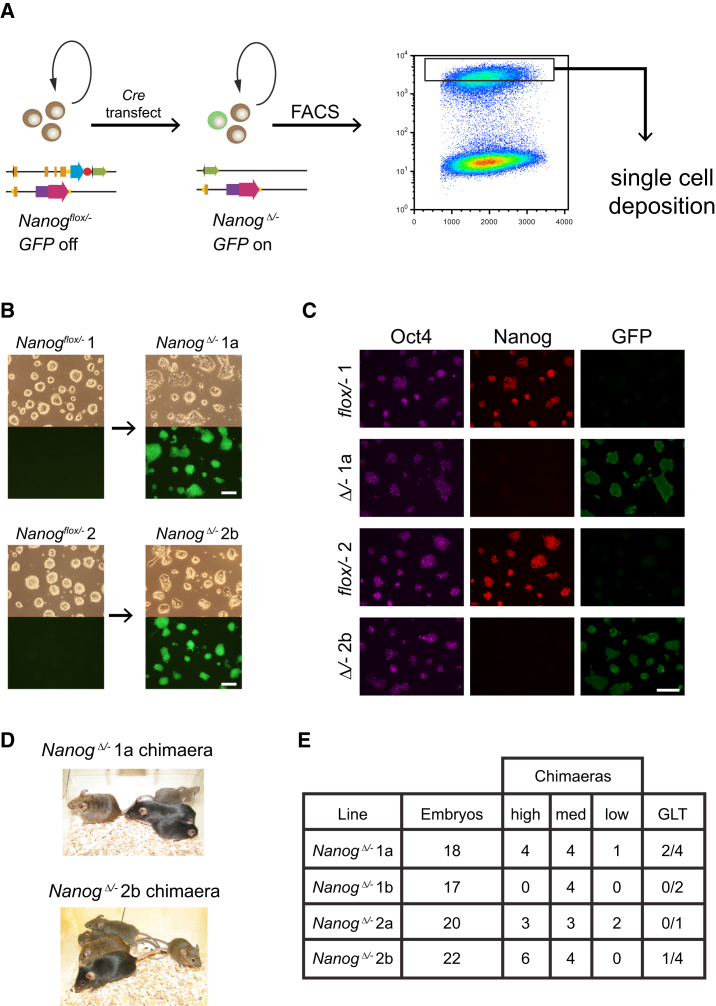
Contribution of *Nanog*-Null ESCs to Adult Chimeras, Including the Germline (A) Strategy for generation *Nanog*^*Δ/−*^ (*Nanog*-null) clonal ESC lines. (B) Phase contrast and fluorescence images of parental and *Nanog*-null ESC lines (scale bar, 100 μm). (C) Oct4, Nanog, and GFP immunostaining of parental and *Nanog*-null ESC lines (scale bar, 100 μm). (D) Chimeras generated from *Nanog*-null ESCs, C57BL/6 mates, and agouti and black pups. High-contribution chimeras generated by injection of agouti *Nanog*-null ESCs into C57BL/6 blastocysts. (E) Summary of blastocyst injections and germline contribution of four clonal *Nanog*-null ESC lines. See also Figures S2 and S3.

The requirement for Nanog in germline development was next assessed using an alternative approach. First, *Nanog*^*flox/−*^ ESC lines were derived from *Nanog*^*flox/flox*^ × *Nanog*^*+/−*^ intercrosses (Figures S2A and S2B). Two independent clones were expanded and exhibited normal ESC morphology (Figure S2C). Both lines gave high contribution chimeras and germline transmission (Figures S2D and S2E). Next, both *Nanog*^*flox/−*^ ESC lines were transiently transfected with Cre, and single GFP-positive cells that had deleted *Nanog* were isolated (Figure 2A). Two GFP-positive clones derived from each parental line were expanded (Figure 2B). All four *Nanog*^*Δ/−*^ clones showed a higher differentiation propensity than parental lines (Figure 2B), consistent with abrogated Nanog function. Successful recombination was confirmed by genomic PCR (Figure S3A). Nanog was undetectable by quantitative real-time PCR (Figure S3B) or immunostaining (Figure 2C). *Nanog*^*Δ/−*^ lines were injected into C57BL/6 blastocysts and three out of four clones produced high contribution coat color chimeras (Figure 2D and 2E). On test crossing, chimeras generated with two independent clones (derived from different parental lines) produced agouti pups, indicating successful germline transmission. This was confirmed by detection of either the null or the deleted band in agouti offspring (Figures 2E and S3D) and detection of GFP fluorescence from the recombined allele in inner cell masses (ICMs) from a further test cross (Figure S3C). These results demonstrate clearly that *Nanog* function is not absolutely required for germline development.

### Esrrb Can Compensate for Nanog Loss in PGCLCs *In Vitro*

**Figure 3:**
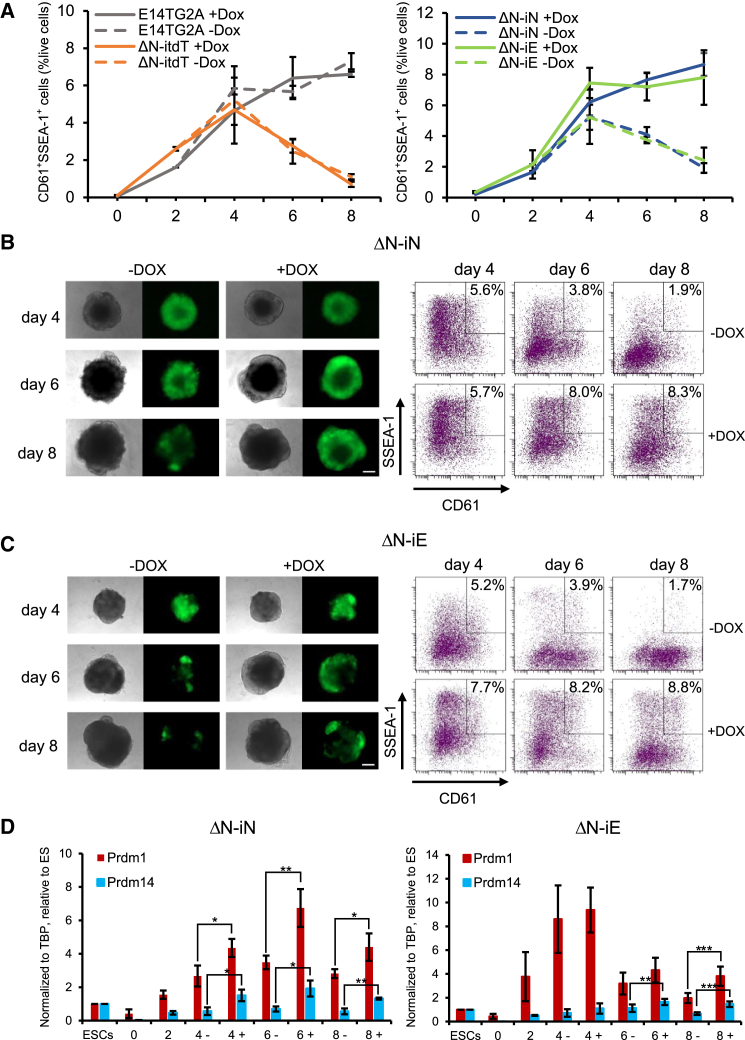
Esrrb Can Replace the Nanog Requirement for Efficient PGCLC Differentiation (A) The proportion of SSEA1^+^/CD61^+^ cells during PGC differentiation of E14TG2A and ΔN-itdT (left) or ΔN-iNanog (ΔN-iN) and ΔN-iEsrrb (ΔN-iE) (right) ESCs are shown at the indicated days of PGCLC differentiation in the absence (−) or presence (+) of Dox addition from day 2 onward (please refer to Figure S5A for differentiation protocol details). Values are means ± SDs; n = 3 biological replicates. (B and C) PGCLC differentiation of ΔN-iN (B) and ΔN-iE (C) ESCs in the presence (+) or absence (−) of Dox. The morphology and Nanog:GFP expression of aggregates are shown (left; scale bar, 200 μm) with SSEA1/CD61 analysis by fluorescence-activated cell sorting (FACS) (right). (D) Quantitative mRNA expression analysis during PGC differentiation of ΔN-iN (left) and ΔN-iE (right) in the presence (+) or absence (−) of Dox at the indicated number of days of PGCLC differentiation. Values are means ± SDs; n = 3 biological replicates. ^∗^p < 0.05; ^∗∗^p < 0.01; and ^∗∗∗^p < 0.001 (unpaired Student’s t test). See also Figures S4–S6.

Early PGC development can be recapitulated *in vitro* by the induction of PGCLCs (Figure S4A) (Hayashi et al., 2011). Naive ESCs in 2 inhibitors (2i)/LIF acquire competence for PGCLC induction after 2 days of culture in basic fibroblast growth factor (bFGF), Activin A, and knockout serum replacement (KSR) (Figures S4A, SD, and SE). Expression of *Prdm1* (also known as *Blimp1*) and *Prdm14,* accompanied by elevated levels of both *Nanog* and *Esrrb* (Figure S4E) indicates PGCLC induction. In keeping with recently published data (Murakami et al., 2016), *Nanog*-null ESCs produced fewer PGCLCs than wild-type controls, as measured by a decrease in CD61^+^/SSEA-1^+^ cells after day 4 (Figure 3A). Next, the doxycycline (Dox)-inducible system for gene expression in *Nanog*-null cells (Festuccia et al., 2012) was assessed for its ability to drive inducible transgene expression during PGCLC differentiation (Figure S4B). The addition of Dox on day 2 allowed robust expression of a tdTomato transgene (Figure S4C) without affecting PGCLC induction efficiency in either wild-type or *Nanog* mutant ESCs (Figures 3A and S4D). The same strategy induced expression of *Nanog* (Figures 3B and S5A) and rescued the deficit in PGCLCs on day 6 and 8 to wild-type levels (Figures 3A and 3B). ESRRB is a downstream mediator of NANOG function in ESCs and during reprogramming (Festuccia et al., 2012). Deletion of *Esrrb* also reduces PGC numbers *in vivo* (Mitsunaga et al., 2004). Interestingly, therefore, Esrrb mRNA was detectable in E14Tg2a and *Nanog*^−/−^ cells at day 2 of PGCLC differentiation (Figure S5B). This expression increased during subsequent days of differentiation in wild-type but not *Nanog*^−/−^ cells (Figure S5B). However, induction of Nanog restored the increasing Esrrb mRNA levels during PGCLC differentiation of *Nanog*^−/−^ cells (Figure S5B). These observations raise the hypothesis that ESRRB might also substitute for NANOG in PGCLCs. Using the same strategy, induced expression of *Esrrb* (Figure S5C) also rescues the CD61/SSEA1 expression deficit to an equivalent degree to Nanog (Figures 3A and 3C). PGCLCs rescued by either *Nanog* or *Esrrb* also express both Prdm1 and Prdm14, confirming their identity (Figure 3D). Compared with wild-type, *Nanog*^−/−^ PGCLCs showed an increased proportion of active caspase-3-positive cells, indicative of apoptosis (Figure S6A). This was restored toward wild-type levels by induction of either Nanog or Esrrb (Figure S6A). Induction of either Nanog or Esrrb also increased the staining by anti-phospho-H3, suggestive of increased proliferation (Figure S6B). These results indicate that *Esrrb* can efficiently rescue the deficit in PGCLC differentiation observed in *Nanog* null ESCs.

### Esrrb Can Compensate for Nanog Loss in PGCs *In Vivo*

**Figure 4:**
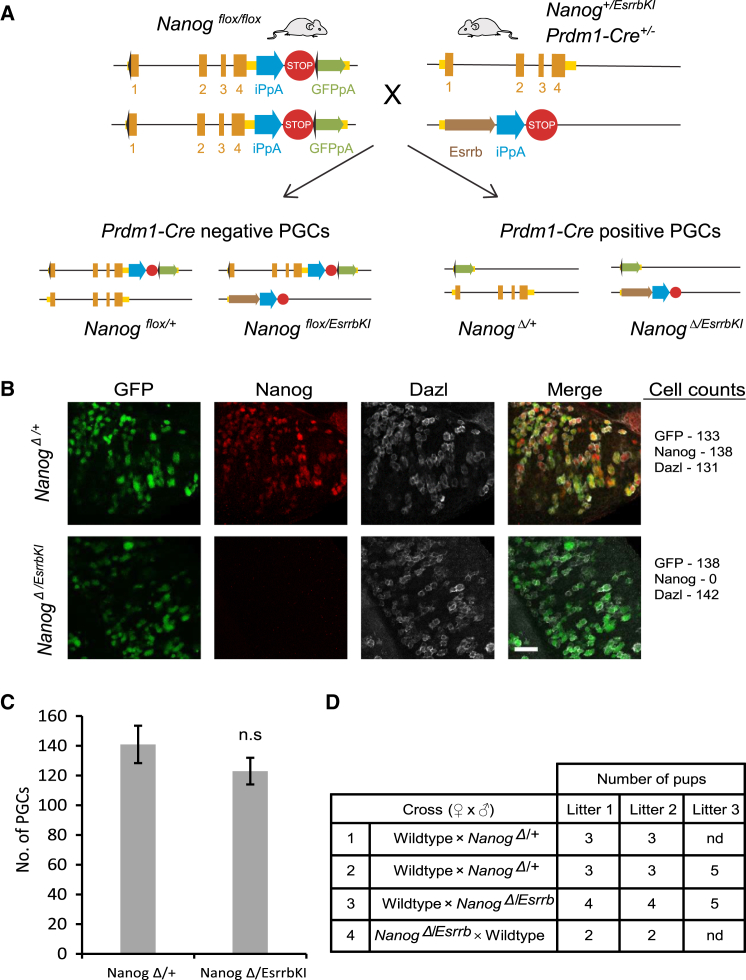
Esrrb Expression Can Rescue Development of *Nanog*^−/−^ PGCs (A) Schematic of *Nanog* conditional knockout, Esrrb knockin strategy. *Nanog*^*flox/flox*^ female mice are crossed with *Prdm1-Cre*: *Nanog*^*+/EsrrbKI*^ male mice. As *Prdm1-Cre* is heterozygous, one in four offspring will be *Nanog* conditional knockout, Esrrb knockin (*Nanog*^*Δ/EsrrbKI*^). (B) E12.5 genital ridges from *Nanog*^*Δ/EsrrbKI*^ and control embryos. GFP expression is from the conditionally deleted (*Δ*) allele and is specific to germ cells of the genital ridge (scale bar, 50 μm). (C) Cell numbers were counted from *Nanog*^*Δ/EsrrbKI*^ and control genital ridges. PGCs are identified by Dazl expression. The mean (± SD) of three biological replicates for control and *Nanog*^*Δ/EsrrbKI*^ are shown. n.s., not significant. (D) Table of breeding data for adult *Nanog*^*Δ/EsrrbKI*^ and control mice. Both male (row 3) and female (row 4) *Nanog*^*Δ/EsrrbKI*^ mice are fertile. See also Figures S7 and S8.

Having established that Esrrb can compensate for Nanog loss in PGCLCs, we next devised a strategy to assess whether Esrrb might compensate for Nanog loss in PGCs *in vivo*. First, ESCs were generated by homologous recombination, in which *Esrrb* cDNA was expressed from the endogenous *Nanog* locus at the Nanog AUG start codon (designated *Esrrb* knockin [KI]) (Figure S7A). Correctly targeted *Nanog*^*+/EsrrbKI*^ ESCs were identified (Figure S7B). To assess Esrrb mRNA expression in the Esrrb knockin model, we analyzed *Nanog*^−/−^ ESCs carrying this Esrrb knockin allele. This showed that *Nanog*^−/−^ ESCs express Esrrb mRNA at ∼60% of the wild-type level and that *Nanog*^*−/EsrrbKI*^ ESCs express Esrrb mRNA at ∼2-fold the level of wild-type ESCs (Figure S7C). *Nanog*^*+/EsrrbKI*^ ESCs were used to establish mouse lines by injection into blastocysts. *Nanog*^*+/EsrrbKI*^ mice were viable and fertile, with no obvious developmental defects (unpublished data). The *Prdm1-Cre-BAC* transgene was then introduced and resulting mice crossed with the *Nanog*^*flox/flox*^ females (Figure 4A). In this case, one in four offspring would carry both an *EssrbKI* and a *Nanog* conditional (*flox*) allele in combination with a *Prdm1-Cre-BAC* transgene (Figure S8A). This combination, which is anticipated to result in *Nanog*^*Δ/EsrrbKI*^ PGCs, was identified by genotyping somatic tissue (Figure S8B). Immunofluorescence of genital ridges from E12.5 *Nanog*^*Δ/EsrrbKI*^ embryos revealed equivalent numbers of DAZL-positive PGCs compared with littermate controls (Figures 4B and 4C). NANOG protein could not be detected in *Nanog*^*Δ/EsrrbKI*^ PGCs, which were instead immunoreactive for GFP (Figure 4B). These results indicate that expression of *Esrrb* under the control of *Nanog* regulatory elements can rescue development of *Nanog*-null PGCs. Furthermore, *Nanog*^*Δ/EsrrbKI*^ PGCs are fully competent to complete germline development, as both male and female of this genotype were fertile (Figures 4D and S8C). Taken together, these observations both *in vitro* and *in vivo* suggest that Esrrb can substitute for Nanog function in germ cells.

## Discussion

Although conditional knockout approaches have shown that the Oct4 and Sox2 are essential for PGC development (Campolo et al., 2013, Kehler et al., 2004), studies on other pluripotency TFs have proved challenging. Here, we use conditional knockout strategies, mouse chimeras, and the recently developed PGCLC system (Hayashi et al., 2011) to assess the function of the Nanog-Esrrb axis in PGCs. This study establishes a role for Nanog in regulating PGC numbers *in vivo*. However, as for ESCs (Chambers et al., 2007), Nanog is strictly dispensable for PGC function. We show that knockin of *Esrrb* to the *Nanog* locus can complement the Nanog defect and is sufficient to rescue PGC numbers *in vivo*. Our study also supports a recently proposed role for Nanog in the maintenance of PGCLCs from pluripotent stem cells *in vitro* (Murakami et al., 2016), but expands on these findings, establishing that Esrrb can substitute for Nanog function in this system. Therefore, in addition to ESC self-renewal and iPSC reprogramming, Esrrb can functionally substitute for Nanog in PGC development. This strengthens the hypothesis that aspects of the naive pluripotency network are re-established in PGCs (Leitch and Smith, 2013). It will therefore be interesting to see whether the recently reported mitotic bookmarking activity of Esrrb in ESCs is also conserved in PGCs (Festuccia et al., 2016).

Previous experiments have suggested that Nanog is required for PGC development (Chambers et al., 2007, Yamaguchi et al., 2009). Although chimera experiments showed that *Nanog*^*−/−*^ ESCs could form nascent PGCs at E11.5, *Nanog*-null PGCs were not observed one day later at E12.5 (Chambers et al., 2007). This loss of PGCs was shown to be due to *Nanog* mutation, since repair of *Nanog* by homologous recombination restored E12.5 PGCs (Chambers et al., 2007). These findings were largely supported by a study in which induced knockdown of Nanog led to PGC death (Yamaguchi et al., 2009). More recently, however, *Nanog*^*−/−*^ iPSCs were reported to be capable of germline transmission based on expression of a GFP transgene in tissues of chimera-derived offspring (Carter et al., 2014). Our present findings provide unequivocal evidence that PGC development can be completed in the absence of Nanog by showing that two newly derived *Nanog*^*−/−*^ ESC lines exhibit germline transmission, as judged by coat color and the presence of *Nanog*-null alleles in F1 pups. Together with the severe reduction in PGC numbers observed in our conditional deletion experiments, this clarifies that the absence of NANOG compromises the development of the PGC population, but that individual PGCs can acquire full functionality in the absence of NANOG. This germline phenotype may render *Nanog*^*−/−*^ PGCs disadvantaged compared with wild-type PGCs in the context of chimeras and reduce the frequency with which germline competency is observed. Our previous *Nanog*-null chimera experiments were performed using ESCs cultured in LIF/fetal calf serum (FCS). In contrast, both current examples of germline transmission were obtained using cells cultured in 2i/LIF, which may have enhanced the degree of chimerism, thereby increasing the likelihood of observing germline transmission, as previously shown for 3i/LIF culture medium (Kiyonari et al., 2010).

The fact that Esrrb can substitute for Nanog provides functional evidence that the naive pluripotency network may be conserved in PGCs. It is of interest that Esrrb fully restored PGC numbers by E12.5 when expressed from *Nanog*. Tetraploid embryos complemented by morula aggregation with *Esrrb*-null ESCs showed a reduction in PGC numbers of 50%–80% between E13.5 and E15.5 (Mitsunaga et al., 2004). Esrrb transcripts were first detected by real-time (RT)-PCR at E11.5 (Mitsunaga et al., 2004), with Nanog expression detected earlier in PGCs (Yamaguchi et al., 2005). However, re-analysis of published single-cell RNA sequencing (RNA-seq) data from PGCs (Hackett et al., 2013, Magnúsdóttir et al., 2013) shows that Esrrb and Nanog mRNAs both increase in expression from E6.5 to E7.5, remaining relatively steady thereafter until E12.5 (Figure S9). This suggests that ESRRB may function in PGCs before E11.5. Esrrb is also expressed during PGCLC differentiation, but at a reduced level in *Nanog*^−/−^ PGCLCs relative to wild-type cells. The wild-type expression level of Esrrb mRNA is restored in *Nanog*^−/−^ PGCLCs by Nanog induction. These results indicate that NANOG controls *Esrrb* expression in PGCs, but that, as is the case in ESCs, positive inputs in addition to NANOG also contribute to *Esrrb* expression (Festuccia et al., 2012, Martello et al., 2012).

*Nanog*^−/−^ cells undergoing PGCLC differentiation showed increased apoptosis and reduced proliferation, validating previous important observations using an *in vivo* conditional knockdown approach (Yamaguchi et al., 2009). Consistent with this seminal study, apoptotic cells positive for active caspase-3 were invariably either OCT4 low or OCT4 negative. Restoring either Nanog or Esrrb expression in *Nanog*^−/−^ PGCLCs is sufficient to reverse both the apoptosis and proliferation defects. Together, these studies add to the evidence that Esrrb is a physiologically relevant mediator of PGCLC function (Mitsunaga et al., 2004).

A limited number of studies have focused on other naive pluripotency factors in the germline. In addition to Oct4 and Sox2, conditional knockout of *Sall4* in PGCs does appear to affect gonadal PGC numbers, although interpretation is complicated by the mosaic deletion brought about by TNAP-Cre (Yamaguchi et al., 2015). The extent to which other pluripotency factors influence germline competence, PGC specification, and subsequent development is of significant interest. The PGCLC system may be an ideal tool to assess these factors. Recently, it was reported that induced expression of Nanog is sufficient to induce PGCLCs from epiblast-like cells (EpiLCs) (Murakami et al., 2016). Together with our data, this may indicate that Nanog has a dose-dependent influence on both the specification and maintenance of PGCs. This is reminiscent of the role of Nanog in ESCs, in which Nanog is not absolutely required, but functions as a pluripotency rheostat (Chambers et al., 2007, Mullin et al., 2017, Mullin et al., 2008). In this regard, it is notable that *Prdm14* is a direct Nanog responsive gene in ESCs (Festuccia et al., 2012, Festuccia et al., 2013) and responds to Nanog in EpiLCs (Murakami et al., 2016). The ability of Esrrb to restore function *in vitro* to *Nanog*^−/−^ PGCLCs further underscores the similarities between naive pluripotency and germline development. It would be interesting to assess whether elevated levels of Nanog or Esrrb *in vivo* might enhance PGC specification and germ cell numbers. How such manipulations of the pluripotency gene regulatory network might affect PGC identity is also of interest. This will enable us to reveal how the pluripotency gene regulatory network interacts with germ-cell-specific genes during PGC development and so build on the remarkably insightful studies that first pioneered the connection between pluripotency and the germline more than half a century ago (Stevens, 1983).

## Experimental Procedures

Animal studies were authorized by a UK Home Office Project License and carried out in a Home-Office-designated facility.

### PGCLC Differentiation

PGCLC differentiation was performed essentially as described previously (Hayashi et al., 2011). Briefly, ESCs were cultured in 2i/LIF medium (as above) for several passages. Cells were then seeded onto fibronectin-coated plates at 1 × 10^5^ cells/12 well in N2B27/1%KSR/bFGF/Activin A to obtain EpiLCs. Two days later, EpiLCs were collected and aggregated at 2,000 cells/well in PGCLC medium (50 ng/mL bone morphogenetic protein (BMP)4, 50 ng/mL BMP8a, 10 ng/mL stem cell factor [SCF], 10 ng/mL epidermal growth factor [EGF], and 1,000 U/mL LIF) using U-bottom 96-well plates (Thermo Fisher Scientific, 174925). For induction of gene expression, 1 μg/mL Dox (Sigma, D9891) was added at day 2 of PGCLC differentiation.

Further methods can be found in Supplemental Experimental Procedures.

## Supplemental Information

Document S1. Supplemental Experimental Procedures, Figures S1–S9, and Tables S1 and S2

Document S2. Article plus Supplemental Information

## References

[bib1] Beddington R.S. (1982). An autoradiographic analysis of tissue potency in different regions of the embryonic ectoderm during gastrulation in the mouse. J. Embryol. Exp. Morphol..

[bib2] Boroviak T., Loos R., Bertone P., Smith A., Nichols J. (2014). The ability of inner-cell-mass cells to self-renew as embryonic stem cells is acquired following epiblast specification. Nat. Cell Biol..

[bib3] Brook F.A., Gardner R.L. (1997). The origin and efficient derivation of embryonic stem cells in the mouse. Proc. Natl. Acad. Sci. USA.

[bib4] Campolo F., Gori M., Favaro R., Nicolis S., Pellegrini M., Botti F., Rossi P., Jannini E.A., Dolci S. (2013). Essential role of Sox2 for the establishment and maintenance of the germ cell line. Stem Cells.

[bib5] Carter A.C., Davis-Dusenbery B.N., Koszka K., Ichida J.K., Eggan K. (2014). Nanog-independent reprogramming to iPSCs with canonical factors. Stem Cell Reports.

[bib6] Chambers I., Silva J., Colby D., Nichols J., Nijmeijer B., Robertson M., Vrana J., Jones K., Grotewold L., Smith A. (2007). Nanog safeguards pluripotency and mediates germline development. Nature.

[bib7] Festuccia N., Osorno R., Halbritter F., Karwacki-Neisius V., Navarro P., Colby D., Wong F., Yates A., Tomlinson S.R., Chambers I. (2012). Esrrb is a direct Nanog target gene that can substitute for Nanog function in pluripotent cells. Cell Stem Cell.

[bib8] Festuccia N., Osorno R., Wilson V., Chambers I. (2013). The role of pluripotency gene regulatory network components in mediating transitions between pluripotent cell states. Curr. Opin. Genet. Dev..

[bib9] Festuccia N., Dubois A., Vandormael-Pournin S., Gallego Tejeda E., Mouren A., Bessonnard S., Mueller F., Proux C., Cohen-Tannoudji M., Navarro P. (2016). Mitotic binding of Esrrb marks key regulatory regions of the pluripotency network. Nat. Cell Biol..

[bib10] Hackett J.A., Sengupta R., Zylicz J.J., Murakami K., Lee C., Down T.A., Surani M.A. (2013). Germline DNA demethylation dynamics and imprint erasure through 5-hydroxymethylcytosine. Science.

[bib11] Hayashi K., Ohta H., Kurimoto K., Aramaki S., Saitou M. (2011). Reconstitution of the mouse germ cell specification pathway in culture by pluripotent stem cells. Cell.

[bib12] Ivanova N., Dobrin R., Lu R., Kotenko I., Levorse J., DeCoste C., Schafer X., Lun Y., Lemischka I.R. (2006). Dissecting self-renewal in stem cells with RNA interference. Nature.

[bib13] Kehler J., Tolkunova E., Koschorz B., Pesce M., Gentile L., Boiani M., Lomelí H., Nagy A., McLaughlin K.J., Schöler H.R., Tomilin A. (2004). Oct4 is required for primordial germ cell survival. EMBO Rep..

[bib14] Kim S., Günesdogan U., Zylicz J.J., Hackett J.A., Cougot D., Bao S., Lee C., Dietmann S., Allen G.E., Sengupta R., Surani M.A. (2014). PRMT5 protects genomic integrity during global DNA demethylation in primordial germ cells and preimplantation embryos. Mol. Cell.

[bib15] Kiyonari H., Kaneko M., Abe S., Aizawa S. (2010). Three inhibitors of FGF receptor, ERK, and GSK3 establishes germline-competent embryonic stem cells of C57BL/6N mouse strain with high efficiency and stability. Genesis.

[bib16] Kurimoto K., Yabuta Y., Ohinata Y., Shigeta M., Yamanaka K., Saitou M. (2008). Complex genome-wide transcription dynamics orchestrated by Blimp1 for the specification of the germ cell lineage in mice. Genes Dev..

[bib17] Leitch H.G., Smith A. (2013). The mammalian germline as a pluripotency cycle. Development.

[bib18] Leitch H.G., Nichols J., Humphreys P., Mulas C., Martello G., Lee C., Jones K., Surani M.A., Smith A. (2013). Rebuilding pluripotency from primordial germ cells. Stem Cell Reports.

[bib19] Leitch H.G., Okamura D., Durcova-Hills G., Stewart C.L., Gardner R.L., Matsui Y., Papaioannou V.E. (2014). On the fate of primordial germ cells injected into early mouse embryos. Dev. Biol..

[bib20] Magnúsdóttir E., Dietmann S., Murakami K., Günesdogan U., Tang F., Bao S., Diamanti E., Lao K., Göttgens B., Azim Surani M. (2013). A tripartite transcription factor network regulates primordial germ cell specification in mice. Nat. Cell Biol..

[bib21] Martello G., Sugimoto T., Diamanti E., Joshi A., Hannah R., Ohtsuka S., Göttgens B., Niwa H., Smith A. (2012). Esrrb is a pivotal target of the Gsk3/Tcf3 axis regulating embryonic stem cell self-renewal. Cell Stem Cell.

[bib22] Matsui Y., Zsebo K., Hogan B.L. (1992). Derivation of pluripotential embryonic stem cells from murine primordial germ cells in culture. Cell.

[bib23] Mitsui K., Tokuzawa Y., Itoh H., Segawa K., Murakami M., Takahashi K., Maruyama M., Maeda M., Yamanaka S. (2003). The homeoprotein Nanog is required for maintenance of pluripotency in mouse epiblast and ES cells. Cell.

[bib24] Mitsunaga K., Araki K., Mizusaki H., Morohashi K., Haruna K., Nakagata N., Giguère V., Yamamura K., Abe K. (2004). Loss of PGC-specific expression of the orphan nuclear receptor ERR-beta results in reduction of germ cell number in mouse embryos. Mech. Dev..

[bib25] Mullin N.P., Yates A., Rowe A.J., Nijmeijer B., Colby D., Barlow P.N., Walkinshaw M.D., Chambers I. (2008). The pluripotency rheostat Nanog functions as a dimer. Biochem. J..

[bib26] Mullin N.P., Gagliardi A., Khoa L.T.P., Colby D., Hall-Ponsele E., Rowe A.J., Chambers I. (2017). Distinct contributions of tryptophan residues within the dimerization domain to Nanog function. J. Mol. Biol..

[bib27] Murakami K., Günesdogan U., Zylicz J.J., Tang W.W.C., Sengupta R., Kobayashi T., Kim S., Butler R., Dietmann S., Surani M.A. (2016). NANOG alone induces germ cells in primed epiblast in vitro by activation of enhancers. Nature.

[bib28] Ohinata Y., Payer B., O’Carroll D., Ancelin K., Ono Y., Sano M., Barton S.C., Obukhanych T., Nussenzweig M., Tarakhovsky A. (2005). Blimp1 is a critical determinant of the germ cell lineage in mice. Nature.

[bib29] Osorno R., Tsakiridis A., Wong F., Cambray N., Economou C., Wilkie R., Blin G., Scotting P.J., Chambers I., Wilson V. (2012). The developmental dismantling of pluripotency is reversed by ectopic Oct4 expression. Development.

[bib30] Resnick J.L., Bixler L.S., Cheng L., Donovan P.J. (1992). Long-term proliferation of mouse primordial germ cells in culture. Nature.

[bib31] Silva J., Nichols J., Theunissen T.W., Guo G., van Oosten A.L., Barrandon O., Wray J., Yamanaka S., Chambers I., Smith A. (2009). Nanog is the gateway to the pluripotent ground state. Cell.

[bib32] Smith A. (2017). Formative pluripotency: the executive phase in a developmental continuum. Development.

[bib33] Stevens L.C., Silver L.M., Martin G.R., Strickland S. (1983). The origin and development of testicular, ovarian, and embryo-derived teratomas. Cold Spring Harbor Conferences on Cell Proliferation.

[bib34] Tam P.P., Zhou S.X. (1996). The allocation of epiblast cells to ectodermal and germ-line lineages is influenced by the position of the cells in the gastrulating mouse embryo. Dev. Biol..

[bib35] Yamaguchi S., Kimura H., Tada M., Nakatsuji N., Tada T. (2005). Nanog expression in mouse germ cell development. Gene Expr. Patterns.

[bib36] Yamaguchi S., Kurimoto K., Yabuta Y., Sasaki H., Nakatsuji N., Saitou M., Tada T. (2009). Conditional knockdown of Nanog induces apoptotic cell death in mouse migrating primordial germ cells. Development.

[bib37] Yamaguchi Y.L., Tanaka S.S., Kumagai M., Fujimoto Y., Terabayashi T., Matsui Y., Nishinakamura R. (2015). Sall4 is essential for mouse primordial germ cell specification by suppressing somatic cell program genes. Stem Cell.

