# Supplementary material for: Esrrb Complementation Rescues Development of Nanog-Null Germ Cells
Source: Cell Rep. 2018 Jan 9;22(2):332–9. doi: 10.1016/j.celrep.2017.12.060 (PMC5775501; doi:10.1016/j.celrep.2017.12.060)
Supplement: Document S1. Supplemental Experimental Procedures, Figures S1–S9, and Tables S1 and S2 [file mmc1.pdf]

Cell Reports, Volume *22*

Supplemental Information

Esrrb Complementation Rescues Development of *Nanog*-Null Germ Cells

Man Zhang, Harry G. Leitch, Walfred W.C. Tang, Nicola Festuccia, Elisa Hall-Ponsele, Jennifer Nichols, M. Azim Surani, Austin Smith, and Ian Chambers

Figure S1.

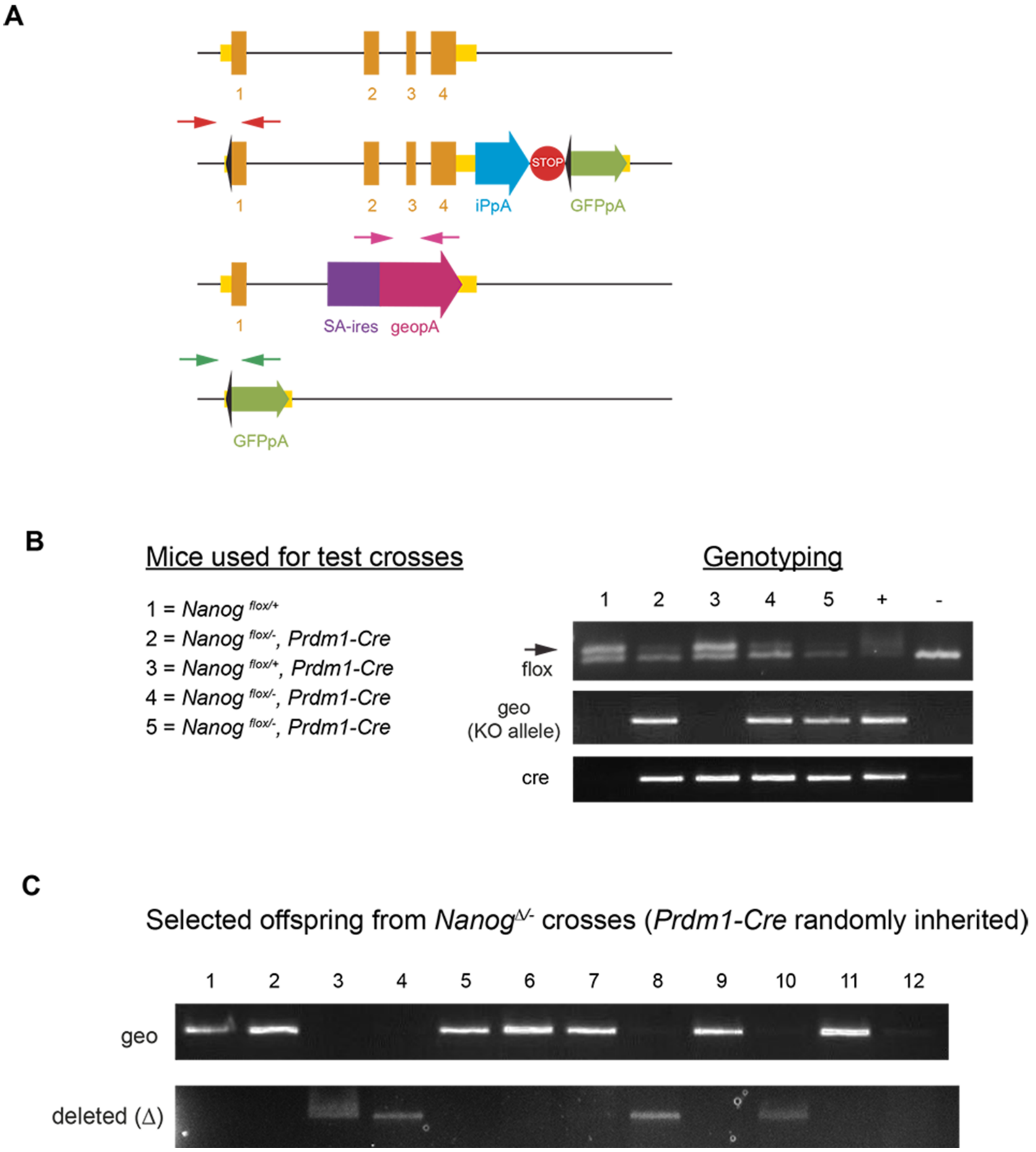

Figure S1. Genotyping of Nanog conditional deletion mice offspring. Related to Figure 1.
(A) Schematic of genotyping strategies.
(B) Genotyping of ear biopsies from adult *Nanog*$^{\Delta/-}$ mice and control crosses.
(C) Genotyping of offspring derived from *Nanog*$^{\Delta/-}$ x wildtype crosses. All offspring carry either the geo (null) or deleted allele.

Figure S2.

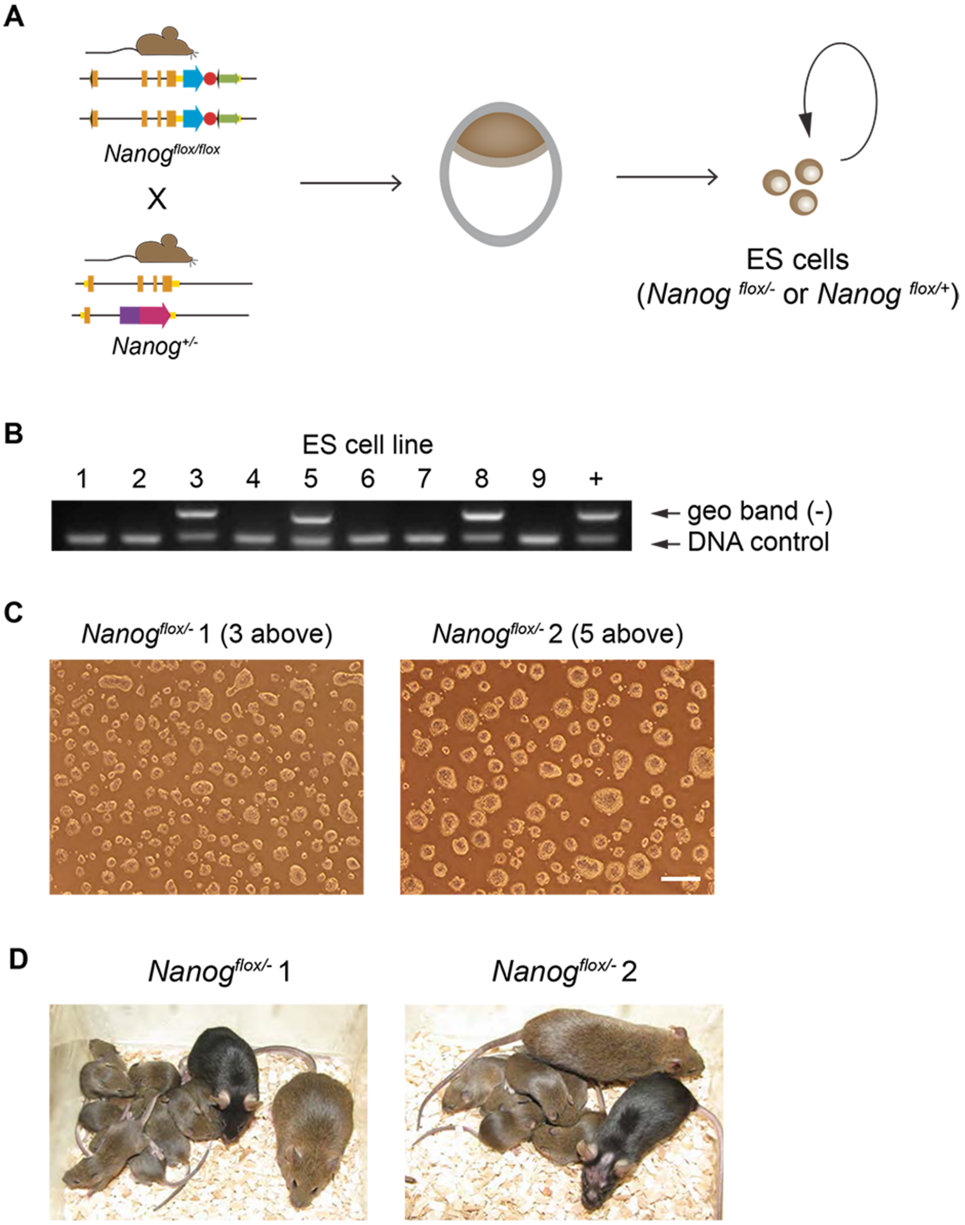

E

		Chimaeras			
Line	Embryos	high	med	low	GLT
Nanog$^{flox/-}$ 1	32	15	0	2	1/3
Nanog$^{flox/-}$ 2	48	23	2	3	1/3

Figure S2. Derivation of germline competent *Nanog*$^{flox/-}$ ESCs. Related to Figure 2.
(A) Strategy for creating *Nanog*$^{flox/-}$ ESCs.
(B) Genotyping of ESCs derived from crosses as in A. Geo band indicates presence of null allele.
(C) Phase/contrast images of two *Nanog*$^{flox/-}$ ESC lines (bar = 100µm).
(D) High contribution coat colour chimaeras (generated by injection of agouti *Nanog*$^{flox/-}$ ESCs into C57BL/6 blastocysts), C57BL/6 mates, and agouti and black pups. Agouti pups indicate germline transmission of *Nanog*$^{flox/-}$ ESCs.
(E) Summary of blastocyst injections and germline transmission of *Nanog* $^{flox/-}$ ESCs.

Figure S3.

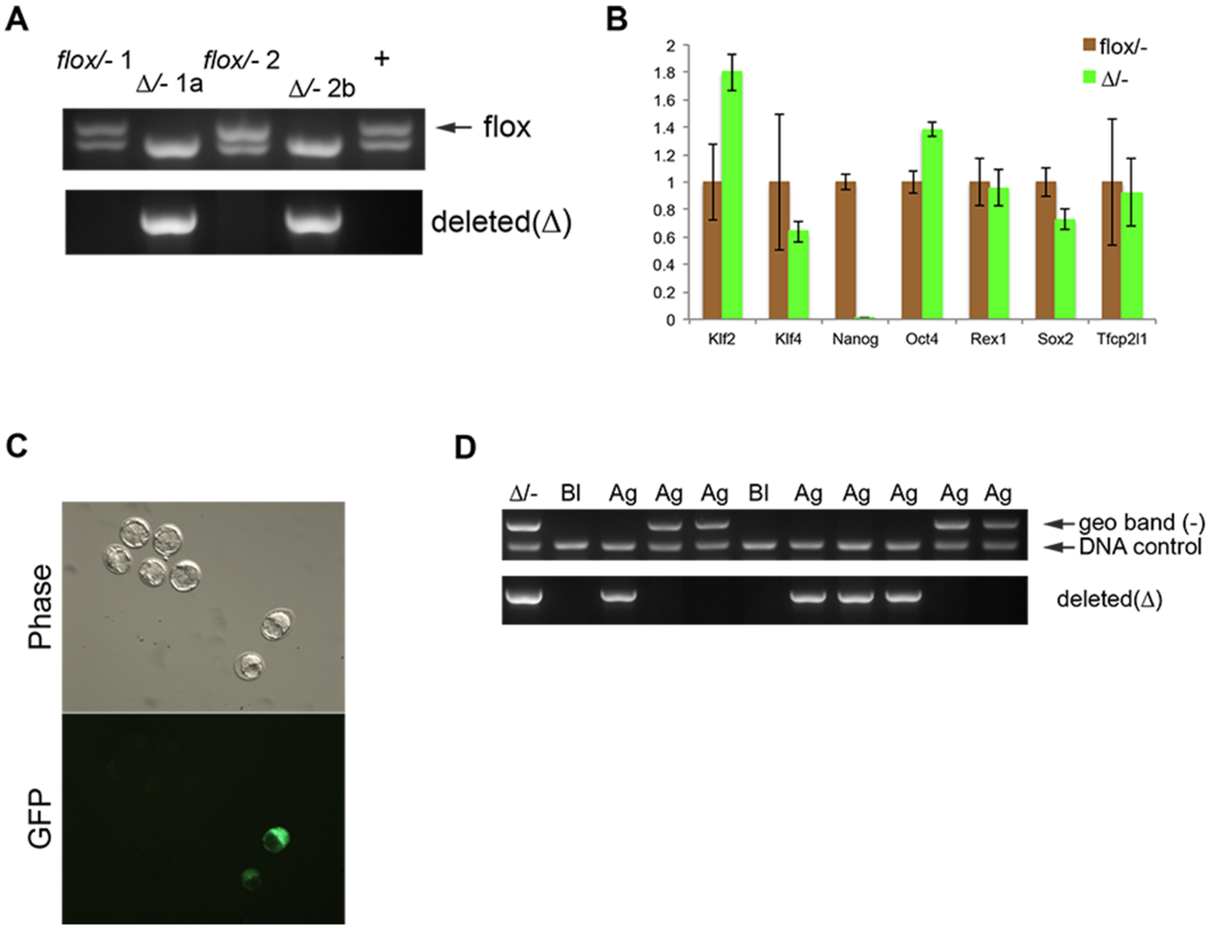

Figure S3. Further characterization of *Nanog*$^{\Delta/-}$ ESCs and their contribution to the germline. Related to Figure 2.
(A) Genotyping of clonal *Nanog*$^{\Delta/-}$ (*Nanog* null) ESC lines.
(B) Quantitative mRNA expression analysis of *Nanog* null and parental (*Nanog*$^{flox/-}$) ESCs. Each genotype is represented by two biological (1a, 1b, 2a, 2b) and two technical replicates. Error bars denote standard deviation.
(C) Phase and fluorescent images of blastocysts derived from chimaera x wildtype matings. Two blastocysts display GFP positive inner cell masses, indicating transmission of the *Nanog* deleted (Δ) allele.
(D) Genotyping of agouti (Ag) and black (Bl) offspring from chimaera x C57Bl/6 crosses. Bl offspring are positive for DNA control only. Ag offspring carry either the null (geo) or deleted (Δ) allele.

Figure S4.

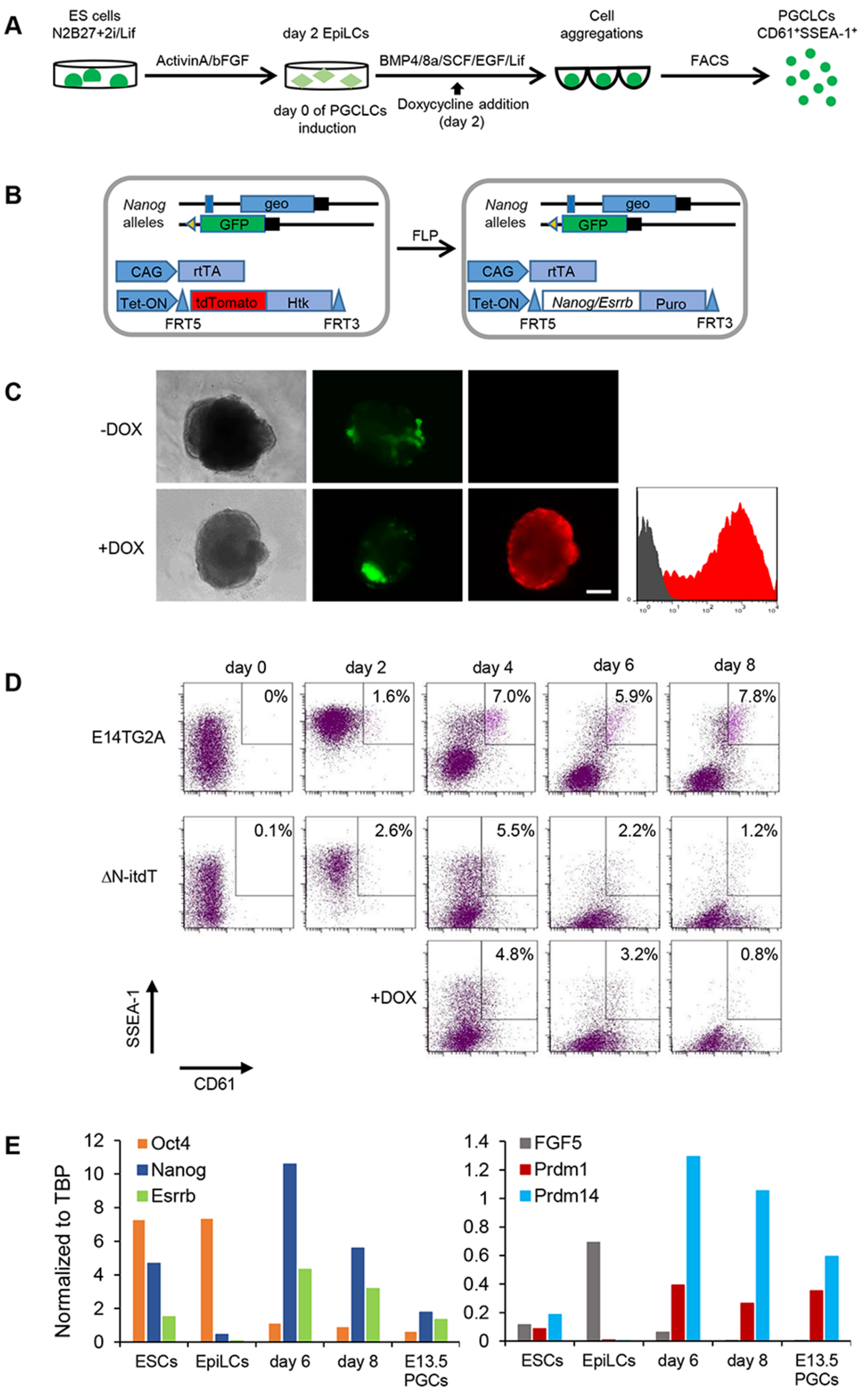

Figure S4. PGC differentiation in vitro. Related to Figure 3.

(A) Scheme for generating PGC like cells (PGCLCs) from ESCs.

(B) The strategy for generating the doxycycline inducible Nanog and Esrrb cell lines from the parental Doxycycline inducible tdTomato (itdT) Nanog null ESC line.

(C) ΔN-itdT cell aggregations, showing morphology, Nanog:GFP expression and FACS analysis of the tdTomato (grey, minus dox; red, plus dox) at in vitro PGC differentiation day8. To induce tdTomato, doxycycline was added at day 2 of PGCLC differentiation; bar = 200μm.

(D) FACS analysis for SSEA-1 and CD61 during E14TG2A and ΔN-itdT PGC differentiation. The percentage of $SSEA1^+/CD61^+$ cells are indicated.

(E) Quantitative mRNA analysis at the indicated days of PGCLC differentiation (day6 and 8 samples were first sorted for SSEA1 and CD61 expression). E13.5 genital ridges provide a control.

Figure S5.

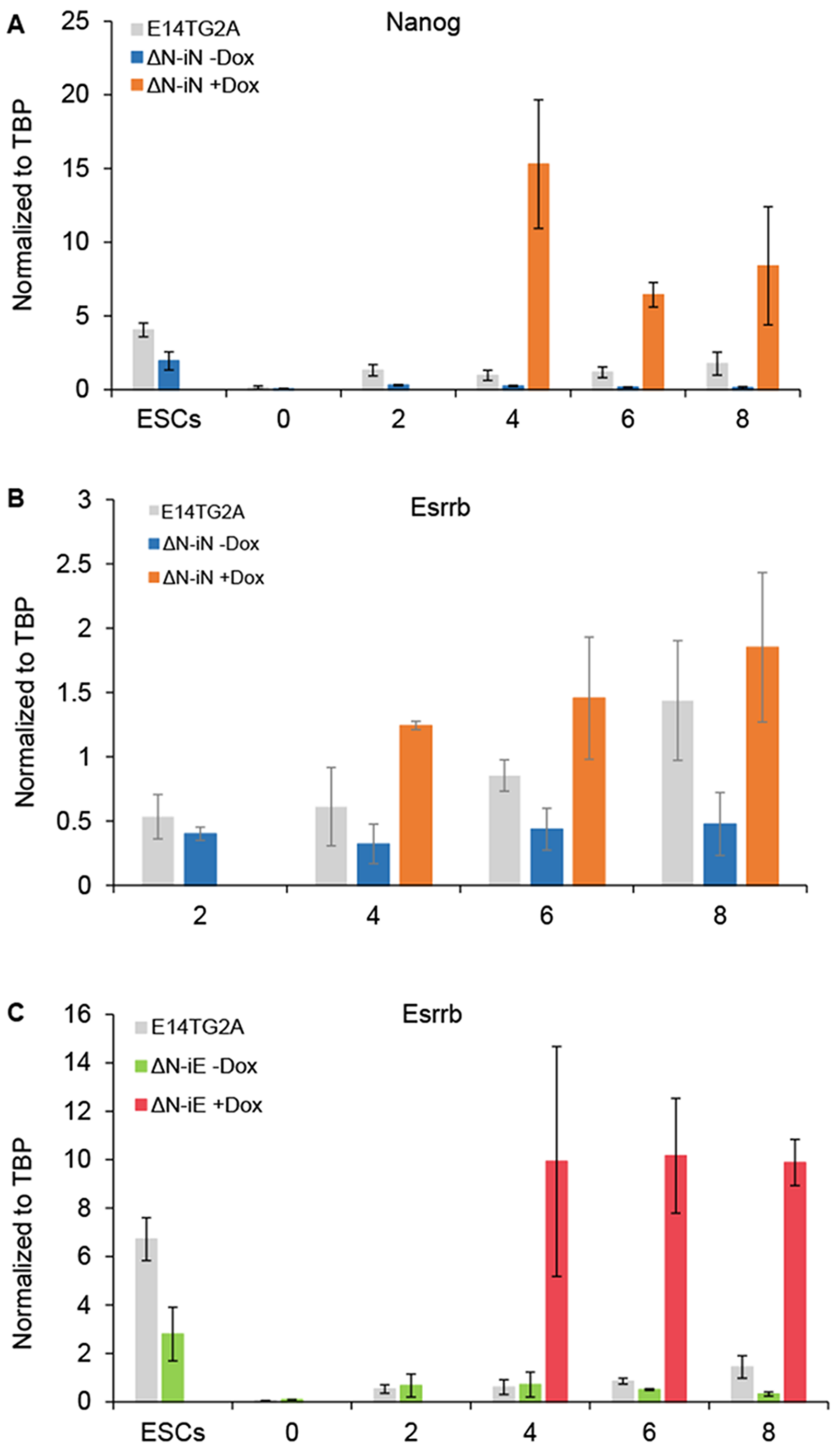

Figure S5. Quantitative transcript analysis during PGCLC differentiation of Nanog-/- derivative lines. Related to Figure 3

(A) Quantitative analysis of Nanog mRNA expression during PGCLC differentiation of wildtype and ΔN-iN cells in the presence or absence of Doxycycline at the indicated number of days. Values are means ± SD; n=3.

(B) Quantitative analysis of Esrrb mRNA expression during PGCLC differentiation of wildtype and ΔN-iN cells in the presence or absence of Doxycycline at the indicated number of days. Values are means ± SD; n=3.

(C) Quantitative analysis of Esrrb mRNA expression during PGCLC differentiation of wildtype and ΔN-iE cells in the presence or absence of Doxycycline at the indicated number of days. Values are means ± SD; n=3.

Figure S6.

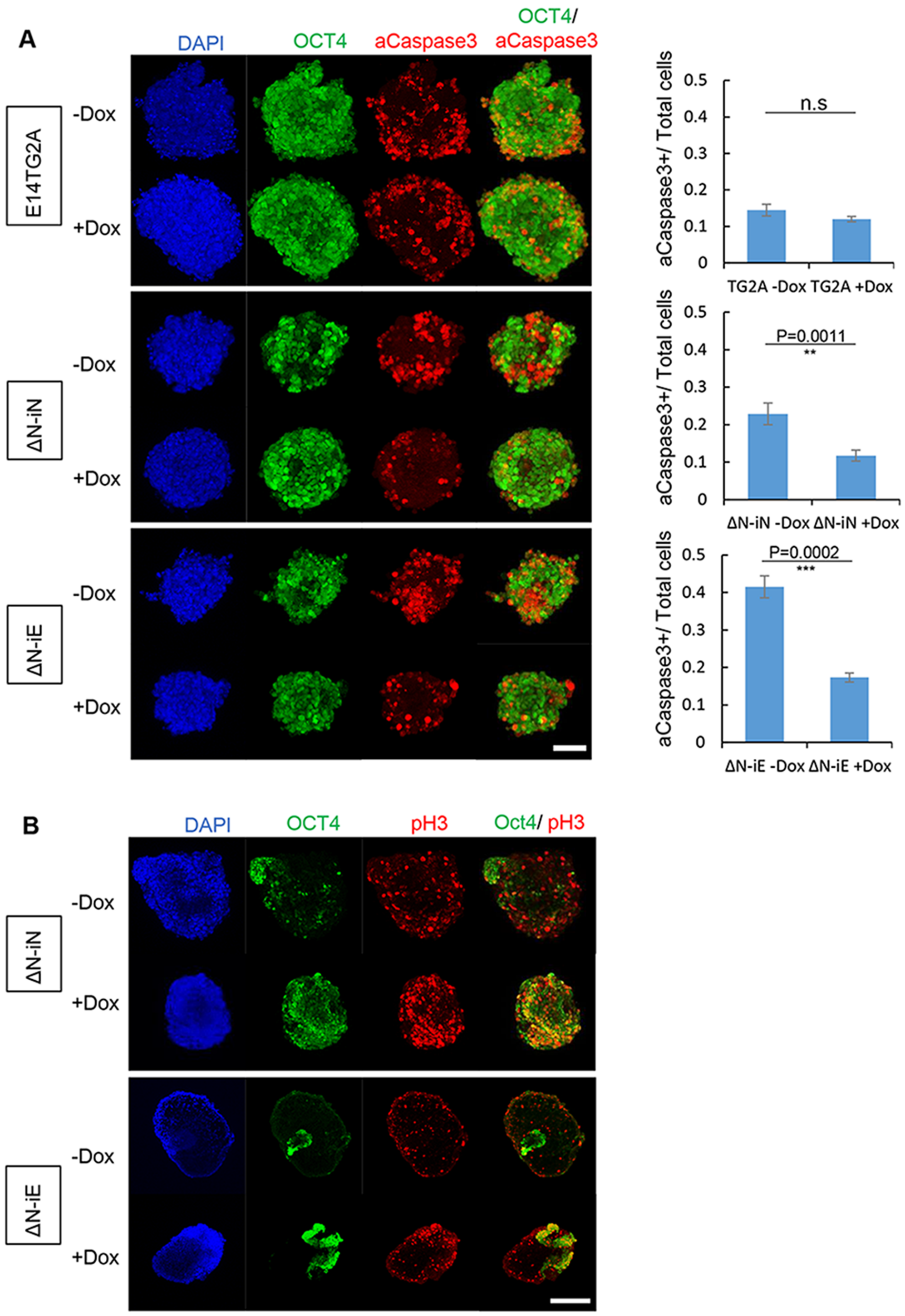

Figure S6. Apoptosis and cell proliferation in PGCLCs. Related to Figure 3.

(A) Confocal images of aggregated SSEA-1^{+}/CD61^{+} cells analysed for OCT4 and active Caspase3 (aCaspase3) (left). Cells were sorted at day 6 of PGCLC differentiation, with Dox added at day 2 as indicated, and cultured for another 2 days. Images are maximum Z-stack projections. Bar=100µm. Quantitition of the proportion of cells positive for active Caspase3 in the population (right): values are means ± SD; n=3; p-values are indicated (unpaired t-test), "n.s", not significant.

(B) Confocal images of day 8 PGCLC aggregations analysed for OCT4 and phospho-Histone 3 (pH3), shown as maximum Z-stack projections. Bar= 200µm.

Figure S7.

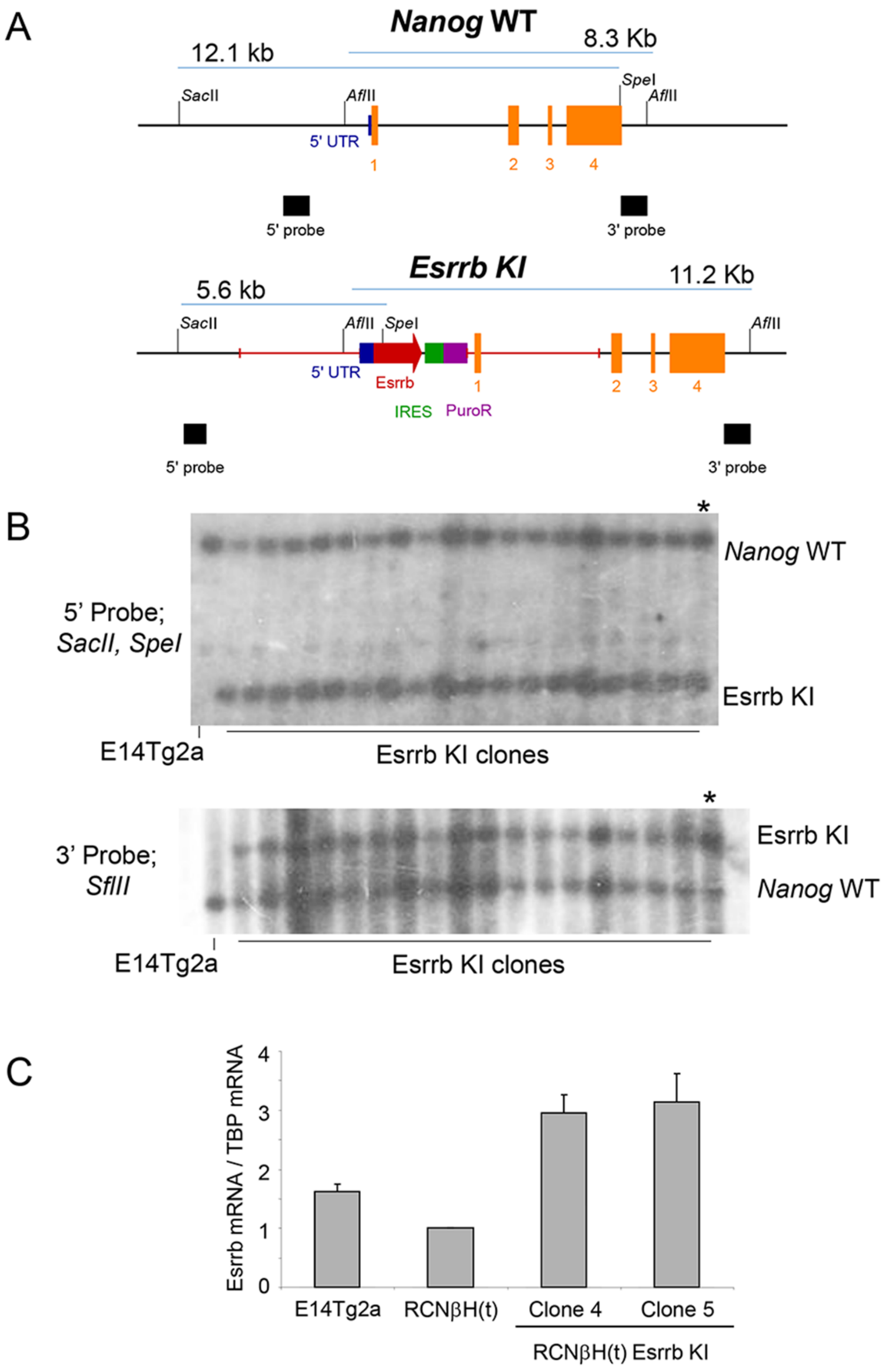

Figure S7. Derivation of E14Tg2a *Nanog*^{EsrrbKI} ESC lines. Related to Figure 4

(A) Schematic representation of the structure of the *Nanog* locus in E14Tg2a and *Nanog*^{EsrrbKI} derivative ESC lines, showing wild-type and targeted *Nanog* alleles, along with restriction sites and DNA probes used for Southern analysis. The expected sizes of the DNA fragments obtained after digestion are shown on top of each diagram. The homology arms of the targeting vector are shown in red.

(B) Southern blots performed on DNA samples from E14Tg2a and puromycin-resistant subclones. *: clone 18 was used to derive mice by blastocyst injection.

(C) Quantitative Esrrb transcript analysis in wild-type, *Nanog*-null and two lines in which one of the *Nanog*-null alleles in RCNβH(t) was rescued by knock-in of the Esrrb targeting vector used in (A).

Figure S8.

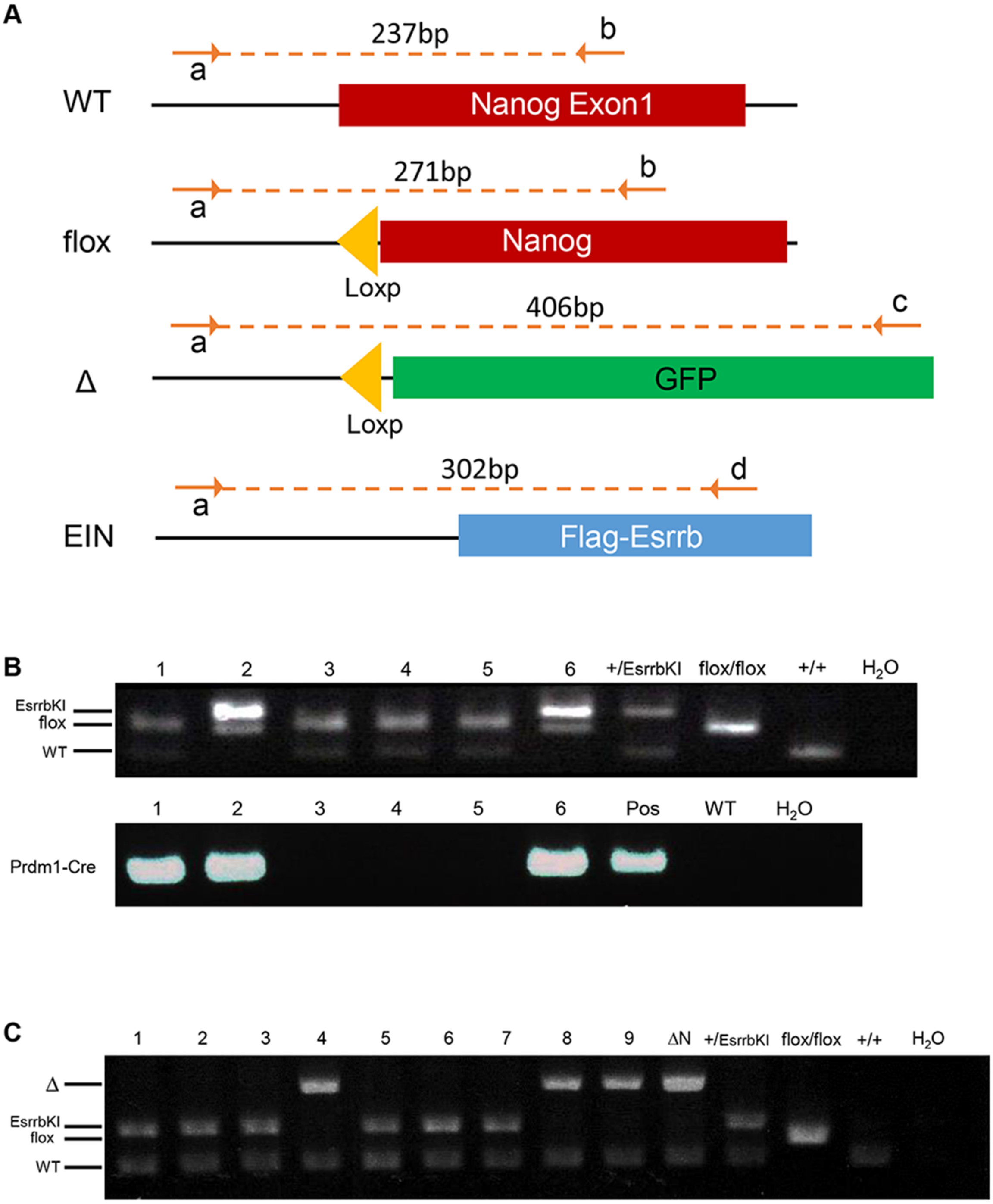

Figure S8. Genotyping of embryos and offspring from crosses of *Nanog*$^{+/EsrrbKI}$ mice. Related to Figure 4.

(A) The genotyping strategies to identify *Nanog* alleles showing wild-type, loxP-flanked alleles before and after excision and Esrrb knock-in.
(B) E12.5 embryos generated by crossing *Nanog*$^{flox/flox}$ female mice with *Prdm1-Cre*: *Nanog*$^{+/EsrrbKI}$ male mice were assessed by PCR. *Nanog*$^{+/EsrrbKI}$, *Nanog*$^{flox/flox}$, *Nanog*$^{+/+}$ DNA samples or H_2O (lanes 7-10) were used as controls.
(C) Genotyping of litters from crosses between adult *Nanog*$^{\Delta/EsrrbKI}$ and wild-type mice. *Nanog*$^{+/\Delta}$ ESCs (ΔN), *Nanog*$^{+/EsrrbKI}$, *Nanog*$^{flox/flox}$, *Nanog*$^{+/+}$ DNA samples or H_2O (lanes 10-14) were used as controls.

Figure S9.

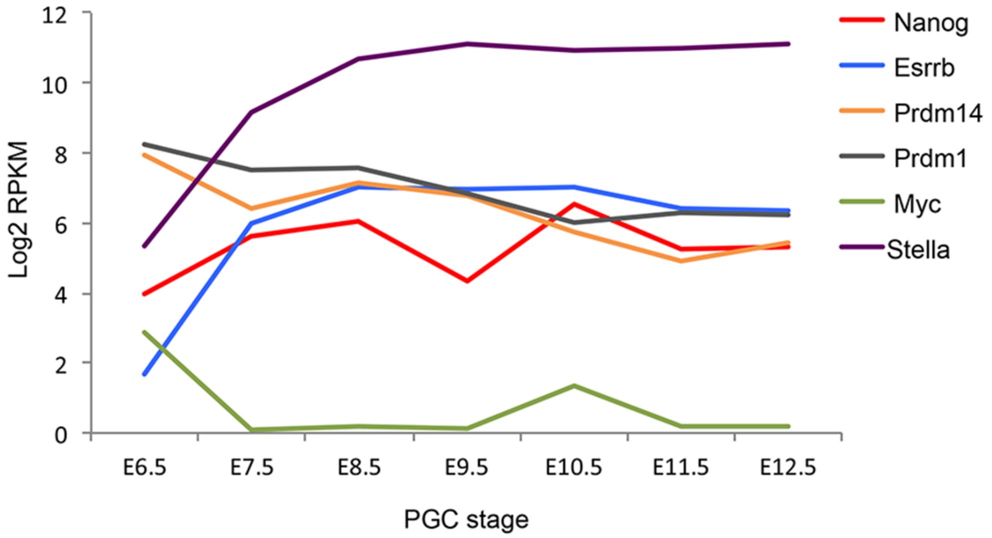

Figure S9. *Esrrb* expression in primordial germ cells. Related to Figures 3 and 4.
Single-cell RNA-seq analysis of Nanog, Esrrb, Prdm1, Prdm15 and Myc expression. Shown as log_2 reads per kilobase of transcript per million mapped reads (RPKM). Primary data from Hackett et al., 2013.

Table S1. All oligonucleotide sequences are given 5' - 3'. Related to Figure 3.

Primers for qPCR	
Primer name	Sequence
Blimp1 Frw	ttctcttggaaaaacgtgtggg
Blimp1 Rev	ggagccggagctagacttg
Prdm14 Frw	tcaattcactcccgaagtacca
Prdm14 Rev	ccggggatggcagaagtaaa
Fgf5 Frw	tgtgtctcaggggattgtagg
Fgf5 Rev	agctgttttcttggaatctctcc
Nanog Frw	tccccacagtttgcctagtt
Nanog Rev	ttctcgggatgaaaaactgc
Oct4 Frw	ttccaccaggcccccc
Oct4 Rev	ggtgagaaggcgaagtctgaag
Esrrb Frw	cgattcatgaaatgcctcaa
Esrrb Rev	cctcctcgaactcggtca
TBP Frw	ggggagctgtgatgtgaagt
TBP Rev	ccaggaaataattctggctca

Table S2. All oligonucleotide sequences are given 5’ -3’.

Primers for Genotyping			
**Primer name**	**Sequence**	**Target(s)**	**Size (bp)**
Prdm1Cre Frw	gccgaggtgcgcgtcagtac	Cre	215
Prdm1Cre Rev	ctgaacatgtccatcaggttcttg		
Nanog locus Frw	gctgcggctcacttccttctgact	WT & flox	237 & 271
Nanog locus Rev1	aggcattgatgaggcgttcccagaatt		
Nanog locus Rev2	gctggatggctccgtcttgatgaa	EsrrbKI	302
Nanog locus Rev3	ggacttgaagaagtcgtgctg	Δ	
Nanog locus Fwd 2	gggtcaccttacagcttcttttgcatta	Deleted	
Nanog locus Rev4	gacttgaagaagtcgtgctgcttcatg		
Bgeo Frw	gttgcagtgcacggcagatacacttgctga	βgeo	
Bgeo Rev	gccactggtgtgggccataattcaattcgc		
TCRD Fwd	caaatgttgcttgtctggtg	DNA (control)	
TCRD Rev	gtcagtcgagtgcacagttt		

Supplemental Experimental Procedures

ESC derivation
Nanog mutant ESC lines were derived as described previously (Nichols et al., 2009). Primary colonies were picked and expanded in 2i/LIF medium (Ying et al., 2008). 2i/LIF medium comprises N2B27 basal medium (Ying et al., 2003) supplemented with 1µM PD0325901, (Axon Medchem), 3 µM CHIR99021 (Axon Medchem) and mouse LIF (prepared in house). Single cell deposition was performed using a MoFlo high-speed cell sorter (Dako Cytomation).

Chimera Production
Chimaeras were produced by microinjection of ESCs (agouti) into C57Bl/6 blastocysts (Nagy et al., 2003).

Immunostaining
For immunostaining of ESCs, cells were fixed in 4% paraformaldehyde (PFA) (10 min, RT) then blocked and permeabilized in PBS/0.1% Triton X-100/1% BSA. Primary antibodies were incubated in the same buffer (overnight, 4C). Secondary antibodies were incubated for 1hr (RT). Cells were washed (3x, 15 min) in PBS after primary and secondary antibody incubations. Nuclei were stained with DAPI. Primary antibodies were: OCT4 (BD, 1:200), NANOG (EBiosciences 1:200) and GFP (abcam, 1:800). Nuclei were stained with DAPI. Alexa Fluor secondary antibodies (Invitrogen) were used at 1:500 dilution.

For wholemount immunostaining, dissected gonads (from E11.5 or E12.5) or PGCLC aggregations were washed twice in PBS containing 3mg/ml polyvinylpyrrolidone (PBS/PVP) , fixed in 4% PFA (20min, RT) and washed three times in PBS/PVP. Samples were then permeabilised in 0.3% Triton X-100 PBS/PVP (45min, RT), blocked (2hours, RT) in PBS / 0.1%BSA / 0.01% Tween20 / 3% goat serum (blocking buffer). Gonads were then incubated with 1:800 chicken anti-GFP antibody (abcam, ab13970), 1:200 rat anti-Nanog antibody (ebioscience, 14-5761-80), 1:100 goat anti-Dazl antibody (Santacruz, sc-27333), 1:500 rabbit anti-DAZL (Abcam ab34139) or 1:500 goat anti-DDX4 (R&D AF2030) diluted in blocking buffer (overnight, 4°C). Aggregations were incubated with either 1:400 rabbit anti-cleaved Caspase3 (Cell signalling, 9661) or 1:200 rabbit anti-phospho Histone 3 (Cell signalling, 53348) combined with 1:200 goat anti-Oct3/4 (Santacruz, sc-8628) diluted in blocking buffer (overnight, 4 °C). The following day, unbound primary antibody was removed by rinsing in 10% blocking buffer/PBS (3×, 15min) and then incubated with the appropriate secondary antibodies. These were 1:500 FITC donkey anti Chicken (abcam), 1:1000 Aldrich 568 Donkey Anti-Rat (sigma, SAB4600077), 1:1000 Alexa Fluor 568 Donkey Anti-rabbit (Invitrogen, A10042) and 1:500 Alexa Fluor 647 Donkey Anti-Goat (Invitrogen, A21447) (3hours, RT). After washing in 10% blocking buffer (3×, 15min), gonads were mounted on glass microscope slides. Aggregations were incubated with DAPI for nuclear staining (overnight, 4°C) and then treated sequentially for 5 mins in 10%, 25%, 50%, 97% thiodiethanol (sigma 166782) before imaging on a Leica SP8 STED-CW Confocal microscope.

FACS analysis
FACS was as described (Zhang et al., 2014). For E14TG2A, ΔN-iN and ΔN-iE cells, Alexa Fluor® 647 anti-CD15(SSEA-1) (Biolegend, 125608) and PE anti-CD61 (Biolegend, 104307) antibodies were used and DAPI was used to gate out dead cells. For ΔN-iTdt cells, Brilliant Violet 421™ anti-CD15 (SSEA-1) (Biolegend, 125613) and APC anti-CD61 (Biolegend, 104315) antibodies were used. The concentration of antibody was tested first. Isotype control antibody Alexa Fluor® 647 Mouse IgM (Biolegend, 401618) and PE Armenian Hamster IgG (Biolegend, 400908) were used to set the gates. Cells were sorted on a BD FACS Aria II and were analyzed on a BD LSR Fortessa (5 laser) analyser.

RNA analysis
Cells were isolated in RLT buffer (Qiagen, 74104) supplemented with 1% β-mercaptoethanol. Cell lysates were homogenized with QIAshredder columns (Qiagen, 79656). Total RNA, free of genomic DNA, was purified using RNeasy mini kit (Qiagen, 74104) and reverse transcribed using the SuperScript® III Reverse Transcriptase kit (Invitrogen, 18080093). Quantitative real-time PCR was done with LightCycler 480 SYBR Green I Master mix (Roche, 04887352001) and LightCycler 480 II machine (Roche, 05015243001). Primers are listed in Table S1. For Nanog null and control ESCs, TaqMan probes for Klf2, Klf4, Nanog, Oct4/Pou5f1, Rex1/Zfp42, Tfcp2l1, Sox2 and Gapdh were used.

Genotyping
Ear notches from juvenile mice, embryos biopsies or ESC pellets were incubated in lysis buffer (1x Qiagen PCR buffer (201205) supplemented with 0.5% Tween 20, 0.5% NP40 and 10µg/ml Proteinase K (Sigma, P2308)) at 56 °C for >3 hours. Proteinase K was then heat-inactivated (95 °C, 10 mins). After centrifugation, 5 µl of lysate was used for genotyping. PCRs were performed with Taq Polymerase (Qiagen 201205) in 30 µl final volume. Primers are listed in Table S2. PCR products were separated on 2% TBE agarose gels and visualised via Ethidium Bromide staining.

Supplemental References

Nichols, J., Silva, J., Roode, M., and Smith, A. (2009). Suppression of Erk signalling promotes ground state pluripotency in the mouse embryo. Development *136*, 3215–3222.

Ying, Q.-L., Stavridis, M., Griffiths, D., Li, M., and Smith, A. (2003). Conversion of embryonic stem cells into neuroectodermal precursors in adherent monoculture. Nat Biotech *21*, 183–186.

Ying, Q.-L., Wray, J., Nichols, J., Batlle-Morera, L., Doble, B., Woodgett, J., Cohen, P., and Smith, A. (2008). The ground state of embryonic stem cell self-renewal. Nature *453*, 519–523.

Zhang, M., Zhou, H., Zheng, C., Xiao, J., Zuo, E., Liu, W., Xie, D., Shi, Y., Wu, C., Wang, H., et al. (2014). The roles of testicular c-kit positive cells in de novo morphogenesis of testis. Sci. Rep. *4*, 5936.
