# Supplementary material for: Esrrb Complementation Rescues Development of Nanog-Null Germ Cells
Source: Cell Rep. 2018 Jan 9;22(2):332–9. doi: 10.1016/j.celrep.2017.12.060 (PMC5775501; doi:10.1016/j.celrep.2017.12.060)
Supplement: Document S2. Article plus Supplemental Information [file mmc2.pdf]

Cell Reports

Report

Esrrb Complementation Rescues Development of *Nanog*-Null Germ Cells

Graphical Abstract

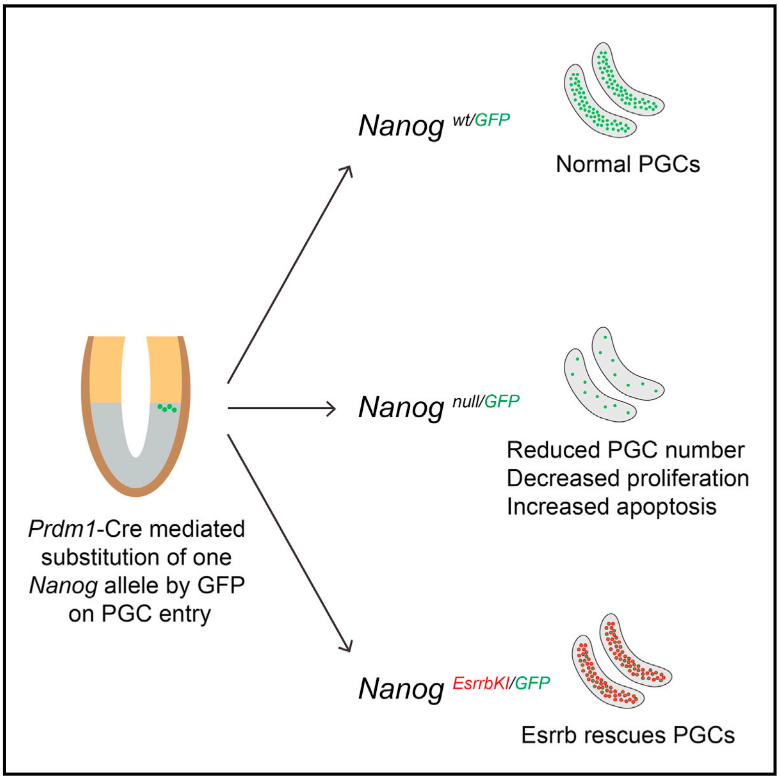

Highlights

- Germline deletion of *Nanog* reduces PGC numbers but does not abolish PGC development
- Without Nanog, PGCLCs form ineffectively with less proliferation and more apoptosis
- The Nanog target gene Esrrb can rescue PGCLC differentiation of *Nanog*$^{-/-}$ ESCs
- Knockin of *Esrrb* at the *Nanog* locus restores PGC development efficiency

Authors

Man Zhang, Harry G. Leitch, Walfred W.C. Tang, ..., M. Azim Surani, Austin Smith, Ian Chambers

Correspondence

h.leitch@lms.mrc.ac.uk (H.G.L.), ichambers@ed.ac.uk (I.C.)

In Brief

Although transcription factors functional in naive pluripotent cells are also expressed in primordial germ cells (PGCs), their PGC role remains unclear. Here, Zhang et al. show that, without *Nanog*, PGCs form ineffectively but that normal PGC development can be restored by induced expression of the NANOG target gene *Esrrb*.

Zhang et al., 2018, Cell Reports *22*, 332–339
January 9, 2018 © 2017 The Author(s).
https://doi.org/10.1016/j.celrep.2017.12.060

CellPress

OPEN ACCESS
CellPress

Cell Reports
Report

Esrrb Complementation Rescues Development of *Nanog*-Null Germ Cells

Man Zhang,[1,7] Harry G. Leitch,[2,6,7,8,9,*] Walfred W.C. Tang,[3,4] Nicola Festuccia,[1,10] Elisa Hall-Ponsele,[1] Jennifer Nichols,[2,4] M. Azim Surani,[3,4] Austin Smith,[2,5] and Ian Chambers[1,6,11,*]
[1]MRC Centre for Regenerative Medicine, Institute for Stem Cell Research, School of Biological Sciences, University of Edinburgh, 5 Little France Drive, Edinburgh EH16 4UU, Scotland
[2]Wellcome Trust-Medical Research Council Stem Cell Institute, University of Cambridge, Cambridge CB2 1QR, United Kingdom
[3]Wellcome Trust/Cancer Research UK Gurdon Institute, Tennis Court Road, University of Cambridge, Cambridge CB2 1QN, United Kingdom
[4]Department of Physiology, Development and Neuroscience, Downing Street, University of Cambridge, Cambridge CB2 3EG, United Kingdom
[5]Department of Biochemistry, University of Cambridge, Tennis Court Road, Cambridge CB2 1GA, United Kingdom
[6]Senior author
[7]These authors contributed equally
[8]Present address: MRC London Institute of Medical Sciences, Du Cane Road, London W12 0NN, England
[9]Present address: Institute of Clinical Sciences, Faculty of Medicine, Imperial College London, Du Cane Road, London W12 0NN, United Kingdom
[10]Present address: Department of Developmental and Stem Cell Biology, Institut Pasteur, CNRS UMR 3738, 25 Rue du Docteur Roux, 75015 Paris, France
[11]Lead Contact
*Correspondence: h.leitch@lms.mrc.ac.uk (H.G.L.), ichambers@ed.ac.uk (I.C.)
https://doi.org/10.1016/j.celrep.2017.12.060

SUMMARY

The transcription factors (TFs) Nanog and Esrrb play important roles in embryonic stem cells (ESCs) and during primordial germ-cell (PGC) development. *Esrrb* is a positively regulated direct target of NANOG in ESCs that can substitute qualitatively for *Nanog* function in ESCs. Whether this functional substitution extends to the germline is unknown. Here, we show that germline deletion of *Nanog* reduces PGC numbers 5-fold at midgestation. Despite this quantitative depletion, *Nanog*-null PGCs can complete germline development in contrast to previous findings. PGC-like cell (PGCLC) differentiation of *Nanog*-null ESCs is also impaired, with *Nanog*-null PGCLCs showing decreased proliferation and increased apoptosis. However, induced expression of Esrrb restores PGCLC numbers as efficiently as Nanog. These effects are recapitulated *in vivo*: knockin of Esrrb to *Nanog* restores PGC numbers to wild-type levels and results in fertile adult mice. These findings demonstrate that Esrrb can replace Nanog function in germ cells.

INTRODUCTION

Naive pluripotency is established in epiblast cells of the mouse blastocyst (Boroviak et al., 2014; Brook and Gardner, 1997). The transcription factors (TFs) *Oct4*, *Sox2*, and *Nanog* are required to establish epiblast identity and are fundamental pluripotency regulators *in vivo* and *in vitro* (Festuccia et al., 2013). Following implantation, the epiblast enters a transitional phase in which cells remain uncommitted and functionally pluripotent (Beddington, 1982; Osorno et al., 2012; Tam and Zhou, 1996). At this point, expression of OCT4 and SOX2, but not NANOG or other naive TFs, is maintained (Smith, 2017). Mouse primordial germ cells (PGCs) are induced from the pluripotent post-implantation epiblast early on embryonic day (E)6 (Ohinata et al., 2005) and upregulate expression of many naive pluripotency genes following specification (Kurimoto et al., 2008). PGCs do not contribute to chimeras when injected into blastocysts (Leitch et al., 2014), but possess a latent capacity to reacquire pluripotency, which can be revealed *in vivo* during teratocarcinogenesis (Stevens, 1983) or by the derivation *in vitro* of naive pluripotent stem cell lines called embryonic germ cells (Leitch et al., 2013; Matsui et al., 1992; Resnick et al., 1992). Furthermore, PGC development is dependent on the expression of pluripotency TFs. Conditional deletion of either *Oct4* or *Sox2* results in PGC death (Campolo et al., 2013; Kehler et al., 2004). *Nanog*-null embryonic stem cells (ESCs) exhibit broad differentiation potential, including to migratory PGCs, but contribution to germ cells at E12.5 was not observed in our previous study (Chambers et al., 2007). Induced knockdown of *Nanog* in PGCs results in significant alteration of their transcriptional program and subsequent apoptosis (Yamaguchi et al., 2009). Induction of PGC-like cells (PGCLCs) *in vitro* is impaired in the absence of *Nanog*, whereas exogenous *Nanog* improves PGCLC yield (Murakami et al., 2016), in keeping with *in vivo* findings.

Nanog is essential for the specification of pluripotency *in vivo* (Mitsui et al., 2003; Silva et al., 2009). However, *Nanog*-null ESCs can be maintained, albeit with a reduced self-renewal efficiency (Chambers et al., 2007). The orphan nuclear receptor Esrrb is a regulator of ESC self-renewal (Festuccia et al., 2012; Ivanova et al., 2006; Martello et al., 2012) and influences PGC numbers *in vivo* (Mitsunaga et al., 2004). *Esrrb* is also a direct NANOG target (Festuccia et al., 2012). Deletion of *Esrrb* abolishes the

 © 2017 The Author(s).
This is an open access article under the CC BY license (http://creativecommons.org/licenses/by/4.0/).

OPEN ACCESS
CellPress

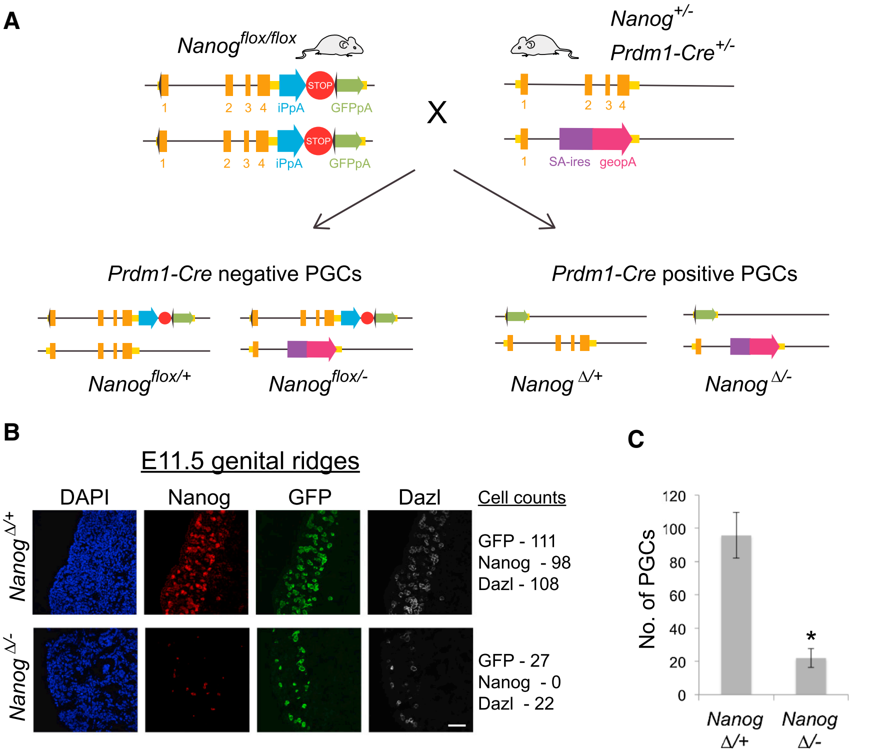

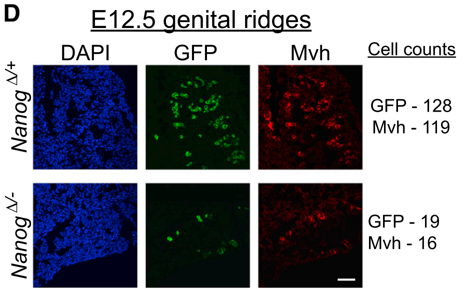

E

	Cross (♀ x ♂)	Number of pups: Litter 1	Litter 2	Litter 3
1	Wildtype x *Nanog*$^{flox/-}$	5	7	4
2	Wildtype x *Nanog*$^{\Delta/-}$	3	3	4
3	*Nanog*$^{\Delta/+}$ x Wildtype	10	nd	nd
4	*Nanog*$^{\Delta/-}$ x Wildtype	5	5	nd
5	*Nanog*$^{\Delta/-}$ x Wildtype	2	3	nd

Figure 1. Conditional Deletion of *Nanog* Reduces PGC Numbers

(A) Strategy for *Nanog* conditional knockout. *Nanog*$^{flox/flox}$ females are crossed with *Nanog*$^{+/-}$; *Prdm1-Cre* male mice. As *Prdm1-Cre* is heterozygous, one in four embryos will have germline deletion of *Nanog* (*Nanog*$^{\Delta/-}$).

(B) E11.5 genital ridge sections from *Nanog*$^{\Delta/-}$ and control embryos immunostained for Nanog, Dazl, and GFP and counterstained with 4',6-diamidino-2-phenylindole (DAPI) (scale bar, 50 μm).

(C) Cell counts of PGCs in *Nanog*$^{\Delta/-}$ and control genital ridges at E11.5. PGCs identified by co-staining for Oct4 and either Dazl or Mvh. The mean (± SD) of two biological and technical replicates for each sample are shown. *$p < 0.05$ (unpaired Student's t test).

(D) E12.5 genital ridges from *Nanog*$^{\Delta/-}$ and control embryos immunostained for GFP and Mvh and counterstained with DAPI (scale bar, 50 μm).

(E) Table of breeding data for adult *Nanog*$^{\Delta/-}$ mice. Both male (row 2) and female (row 4 and 5) *Nanog*$^{\Delta/-}$ mice are fertile.

See also Figure S1.

ability of NANOG to confer leukemia inhibitory factor (LIF) independence in ESCs (Festuccia et al., 2012). Furthermore, ESRRB can compensate for NANOG function in epiblast stem cell (EpiSC) reprogramming and in induced pluripotent stem cell (iPSC) generation (Festuccia et al., 2012). Thus, Esrrb is a key downstream mediator of Nanog function in the maintenance and establishment of pluripotency *in vitro*. Here, we reassess the requirement for Nanog in PGCs and investigate whether ESRRB can compensate for NANOG function during PGC development.

RESULTS

Conditional Deletion of Nanog Reduces PGC Numbers

To assess whether Nanog is required cell autonomously in PGCs, a conditional knockout strategy was used. Mice homozygous for a *Nanog* conditional allele (*Nanog*$^{flox/flox}$) (Chambers et al., 2007) were crossed with *Nanog* heterozygous mice (*Nanog*$^{+/-}$) (Mitsui et al., 2003) harboring the *Prdm1-Cre-BAC* transgene (Ohinata et al., 2005) (Figure 1A). One in four offspring carried the *Nanog* null (−) and conditionally deleted (Δ) alleles in PGCs. As Prdm1-Cre-mediated excision has been reported to be incomplete until after E10.5 (Campolo et al., 2013; Kim et al., 2014), genital ridges in control and mutant embryos were dissected at E11.5 and analyzed by immunofluorescence. NANOG protein was not detected in mutant genital ridges (Figure 1B). However, GFP-positive cells were present, indicating successful deletion of *Nanog* (Figure 1B). GFP-positive cells were positive for DAZL, indicating that these represent *Nanog*-null PGCs (Figure 1B). Compared with littermate controls, PGC numbers in *Nanog* mutant embryos were reduced 80% (Figure 1B and 1C). Surprisingly, a small number of GFP-positive mutant PGCs expressing MVH were also detected at E12.5 (Figure 1D). To establish whether these surviving *Nanog* mutant PGCs were developmentally competent, subsequent litters were allowed to go to term, and adult mutant mice of both sexes were test-crossed (Figures S1A and S1B). Male and female mutant mice were fertile (Figure 1E), passing either the knockout or conditionally deleted allele to their offspring (Figure S1C). These findings indicate that Prdm1-Cre-mediated deletion of *Nanog* reduces the PGC number, but suggest that *Nanog* might not be strictly required for germline development.

Nanog Is Not Essential for Germline Development

The requirement for Nanog in germline development was next assessed using an alternative approach. First, *Nanog*$^{flox/-}$ ESC

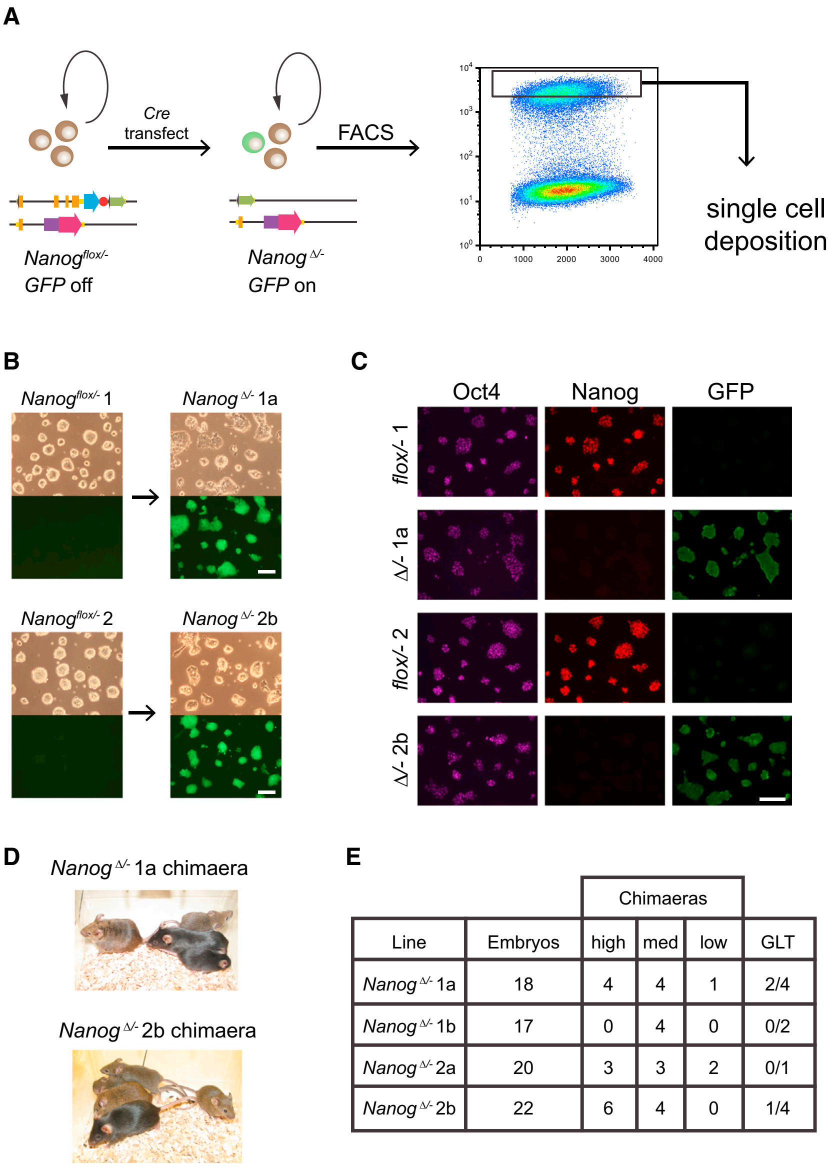

		Chimaeras			
Line	Embryos	high	med	low	GLT
Nanog ^Δ/- 1a	18	4	4	1	2/4
Nanog ^Δ/- 1b	17	0	4	0	0/2
Nanog ^Δ/- 2a	20	3	3	2	0/1
Nanog ^Δ/- 2b	22	6	4	0	1/4

Figure 2. Contribution of *Nanog*-Null ESCs to Adult Chimeras, Including the Germline

(A) Strategy for generation $Nanog^{\Delta/-}$ (*Nanog*-null) clonal ESC lines.

(B) Phase contrast and fluorescence images of parental and *Nanog*-null ESC lines (scale bar, 100 μm).

(C) Oct4, Nanog, and GFP immunostaining of parental and *Nanog*-null ESC lines (scale bar, 100 μm).

(D) Chimeras generated from *Nanog*-null ESCs, C57BL/6 mates, and agouti and black pups. High-contribution chimeras generated by injection of agouti *Nanog*-null ESCs into C57BL/6 blastocysts.

(E) Summary of blastocyst injections and germline contribution of four clonal *Nanog*-null ESC lines.

See also Figures S2 and S3.

lines were derived from $Nanog^{flox/flox}$ × $Nanog^{+/-}$ intercrosses (Figures S2A and S2B). Two independent clones were expanded and exhibited normal ESC morphology (Figure S2C). Both lines gave high contribution chimeras and germline transmission (Figures S2D and S2E). Next, both $Nanog^{flox/-}$ ESC lines were transiently transfected with Cre, and single GFP-positive cells that had deleted *Nanog* were isolated (Figure 2A). Two GFP-positive clones derived from each parental line were expanded (Figure 2B). All four $Nanog^{\Delta/-}$ clones showed a higher differentiation propensity than parental lines (Figure 2B), consistent with abrogated Nanog function. Successful recombination was confirmed by genomic PCR (Figure S3A). Nanog was undetectable by quantitative real-time PCR (Figure S3B) or immunostaining (Figure 2C). $Nanog^{\Delta/-}$ lines were injected into C57BL/6 blastocysts and three out of four clones produced high contribution coat color chimeras (Figure 2D and 2E). On test crossing, chimeras generated with two independent clones (derived from different parental lines) produced agouti pups, indicating successful germline transmission. This was confirmed by detection of either the null or the deleted band in agouti offspring (Figures 2E and S3D) and detection of GFP fluorescence from the recombined allele in inner cell masses (ICMs) from a further test cross (Figure S3C). These results demonstrate clearly that *Nanog* function is not absolutely required for germline development.

Esrrb Can Compensate for Nanog Loss in PGCLCs *In Vitro*

Early PGC development can be recapitulated *in vitro* by the induction of PGCLCs (Figure S4A) (Hayashi et al., 2011). Naive ESCs in 2 inhibitors (2i)/LIF acquire competence for PGCLC induction after 2 days of culture in basic fibroblast growth factor (bFGF), Activin A, and knockout serum replacement (KSR) (Figures S4A, SD, and SE). Expression of *Prdm1* (also known as *Blimp1*) and *Prdm14,* accompanied by elevated levels of both *Nanog* and *Esrrb* (Figure S4E) indicates PGCLC induction. In keeping with recently published data (Murakami et al., 2016), *Nanog*-null ESCs produced fewer PGCLCs than wild-type controls, as measured by a decrease in $CD61^{+}$/SSEA-1^{+} cells after day 4 (Figure 3A). Next, the doxycycline (Dox)-inducible system for gene expression in *Nanog*-null cells (Festuccia et al., 2012) was assessed for its ability to drive inducible transgene expression during PGCLC differentiation (Figure S4B). The addition of Dox on day 2 allowed robust expression of a tdTomato transgene (Figure S4C) without affecting PGCLC induction efficiency in either wild-type or *Nanog* mutant ESCs (Figures 3A and S4D). The same strategy induced expression of *Nanog* (Figures 3B and S5A) and rescued the deficit in PGCLCs on day 6 and 8 to wild-type levels (Figures 3A and 3B). ESRRB is a downstream mediator of NANOG function in ESCs and during reprogramming

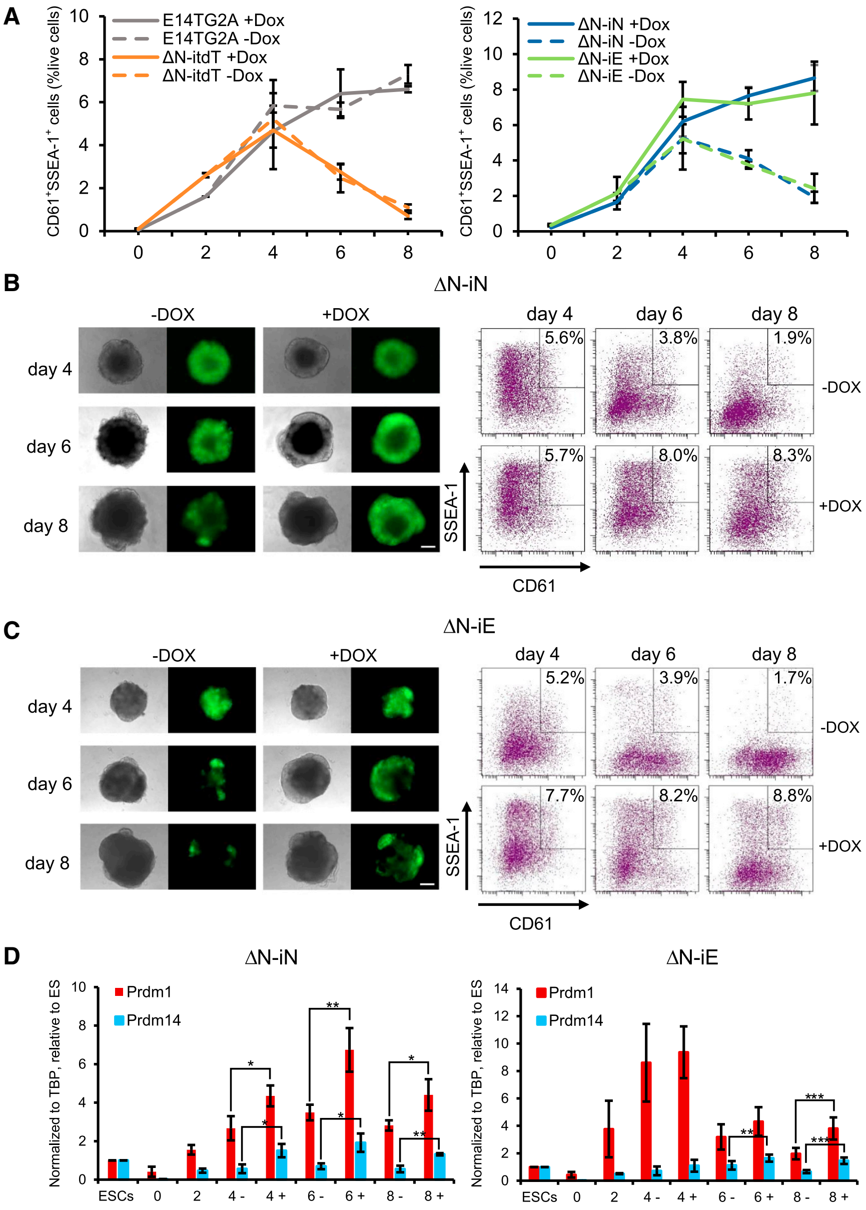

Figure 3. Esrrb Can Replace the Nanog Requirement for Efficient PGCLC Differentiation

(A) The proportion of SSEA1+/CD61+ cells during PGC differentiation of E14TG2A and ΔN-itdT (left) or ΔN-iNanog (ΔN-iN) and ΔN-iEsrrb (ΔN-iE) (right) ESCs are shown at the indicated days of PGCLC differentiation in the absence (–) or presence (+) of Dox addition from day 2 onward (please refer to Figure S5A for differentiation protocol details). Values are means ± SDs; n = 3 biological replicates.

(B and C) PGCLC differentiation of ΔN-iN (B) and ΔN-iE (C) ESCs in the presence (+) or absence (–) of Dox. The morphology and Nanog:GFP expression of aggregates are shown (left; scale bar, 200 μm) with SSEA1/CD61 analysis by fluorescence-activated cell sorting (FACS) (right).

(D) Quantitative mRNA expression analysis during PGC differentiation of ΔN-iN (left) and ΔN-iE (right) in the presence (+) or absence (–) of Dox at the indicated number of days of PGCLC differentiation. Values are means ± SDs; n = 3 biological replicates. *p < 0.05; **p < 0.01; and ***p < 0.001 (unpaired Student's t test).

See also Figures S4–S6.

(Festuccia et al., 2012). Deletion of *Esrrb* also reduces PGC numbers *in vivo* (Mitsunaga et al., 2004). Interestingly, therefore, Esrrb mRNA was detectable in E14Tg2a and *Nanog*$^{-/-}$ cells at day 2 of PGCLC differentiation (Figure S5B). This expression increased during subsequent days of differentiation in wild-type but not *Nanog*$^{-/-}$ cells (Figure S5B). However, induction of Nanog restored the increasing Esrrb mRNA levels during PGCLC differentiation of *Nanog*$^{-/-}$ cells (Figure S5B). These observations raise the hypothesis that ESRRB might also substitute for NANOG in PGCLCs. Using the same strategy, induced expression of *Esrrb* (Figure S5C) also rescues the CD61/SSEA1 expression deficit to an equivalent degree to Nanog (Figures 3A and 3C). PGCLCs rescued by either *Nanog* or *Esrrb* also express both Prdm1 and Prdm14, confirming their identity (Figure 3D). Compared with wild-type, *Nanog*$^{-/-}$ PGCLCs showed an increased proportion of active caspase-3-positive cells, indicative of apoptosis (Figure S6A). This was restored toward wild-type levels by induction of either Nanog or Esrrb (Figure S6A). Induction of either Nanog or Esrrb also increased the staining by anti-phospho-H3, suggestive of increased proliferation (Figure S6B). These results indicate that *Esrrb* can efficiently rescue the deficit in PGCLC differentiation observed in *Nanog* null ESCs.

Esrrb Can Compensate for Nanog Loss in PGCs *In Vivo*

Having established that Esrrb can compensate for Nanog loss in PGCLCs, we next devised a strategy to assess whether Esrrb might compensate for Nanog loss in PGCs *in vivo*. First, ESCs were generated by homologous recombination, in which *Esrrb* cDNA was expressed from the endogenous *Nanog* locus at the Nanog AUG start codon (designated *Esrrb* knockin [KI]) (Figure S7A). Correctly targeted *Nanog*$^{+/EsrrbKI}$ ESCs were identified (Figure S7B). To assess Esrrb mRNA expression in the Esrrb knockin model, we analyzed *Nanog*$^{-/-}$ ESCs carrying this Esrrb knockin allele. This showed that *Nanog*$^{-/-}$ ESCs express Esrrb mRNA at ~60% of the wild-type level and that *Nanog*$^{-/EsrrbKI}$ ESCs express Esrrb mRNA at ~2-fold the level of wild-type ESCs (Figure S7C). *Nanog*$^{+/EsrrbKI}$ ESCs were used to establish mouse lines by injection into blastocysts. *Nanog*$^{+/EsrrbKI}$ mice were viable and fertile, with no obvious developmental defects (unpublished data). The *Prdm1-Cre-BAC* transgene was then

OPEN ACCESS
CellPress

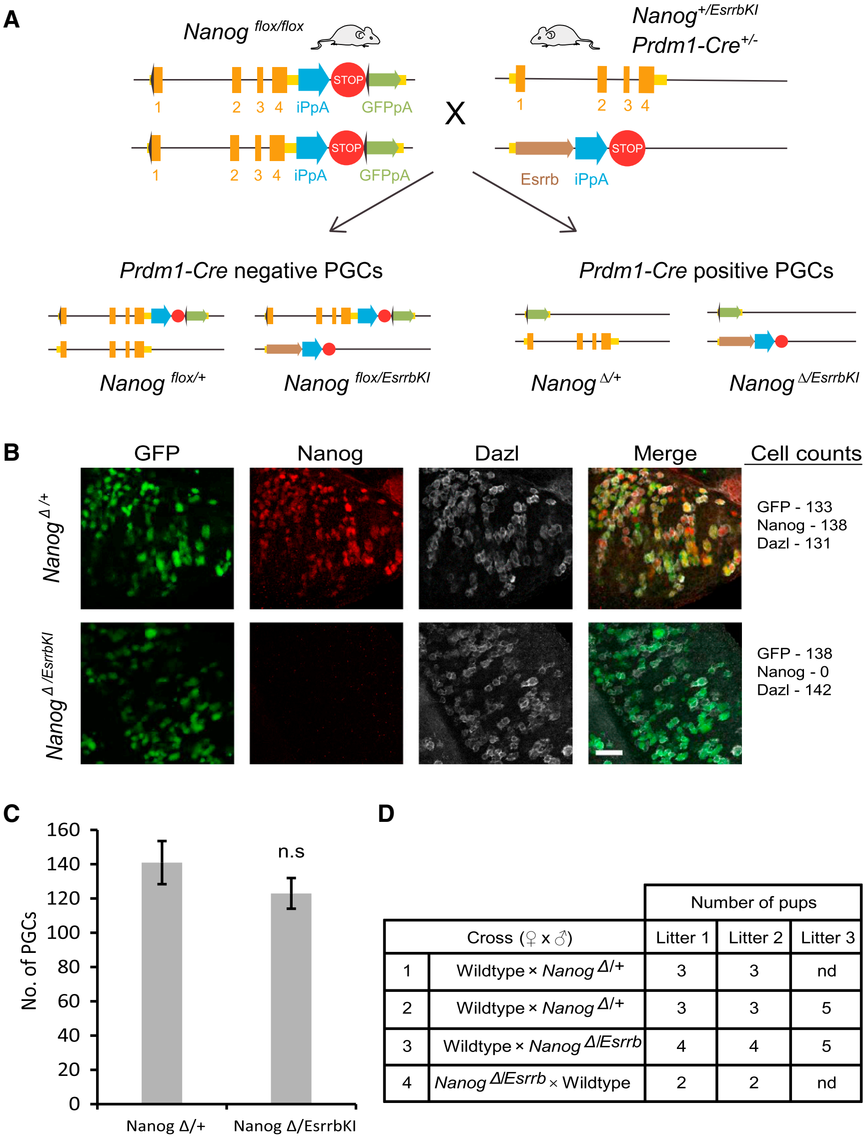

	Cross (♀ x ♂)	Number of pups Litter 1	Litter 2	Litter 3
1	Wildtype × Nanog $^{\Delta/+}$	3	3	nd
2	Wildtype × Nanog $^{\Delta/+}$	3	3	5
3	Wildtype × Nanog $^{\Delta/Esrrb}$	4	4	5
4	Nanog $^{\Delta/Esrrb}$ × Wildtype	2	2	nd

Figure 4. Esrrb Expression Can Rescue Development of *Nanog*$^{-/-}$ PGCs

(A) Schematic of *Nanog* conditional knockout, Esrrb knockin strategy. *Nanog*$^{flox/flox}$ female mice are crossed with *Prdm1-Cre*: *Nanog*$^{+/EsrrbKI}$ male mice. As *Prdm1-Cre* is heterozygous, one in four offspring will be *Nanog* conditional knockout, Esrrb knockin (*Nanog*$^{\Delta/EsrrbKI}$).

(B) E12.5 genital ridges from *Nanog*$^{\Delta/EsrrbKI}$ and control embryos. GFP expression is from the conditionally deleted (Δ) allele and is specific to germ cells of the genital ridge (scale bar, 50 μm).

(C) Cell numbers were counted from *Nanog*$^{\Delta/EsrrbKI}$ and control genital ridges. PGCs are identified by Dazl expression. The mean (± SD) of three biological replicates for control and *Nanog*$^{\Delta/EsrrbKI}$ are shown. n.s., not significant.

(D) Table of breeding data for adult *Nanog*$^{\Delta/EsrrbKI}$ and control mice. Both male (row 3) and female (row 4) *Nanog*$^{\Delta/EsrrbKI}$ mice are fertile.

See also Figures S7 and S8.

introduced and resulting mice crossed with the *Nanog*$^{flox/flox}$ females (Figure 4A). In this case, one in four offspring would carry both an *EssrbKI* and a *Nanog* conditional (*flox*) allele in combination with a *Prdm1-Cre-BAC* transgene (Figure S8A). This combination, which is anticipated to result in *Nanog*$^{\Delta/EsrrbKI}$ PGCs, was identified by genotyping somatic tissue (Figure S8B). Immunofluorescence of genital ridges from E12.5 *Nanog*$^{\Delta/EsrrbKI}$ embryos revealed equivalent numbers of DAZL-positive PGCs compared with littermate controls (Figures 4B and 4C). NANOG protein could not be detected in *Nanog*$^{\Delta/EsrrbKI}$ PGCs, which were instead immunoreactive for GFP (Figure 4B). These results indicate that expression of *Esrrb* under the control of *Nanog* regulatory elements can rescue development of *Nanog*-null PGCs. Furthermore, *Nanog*$^{\Delta/EsrrbKI}$ PGCs are fully competent to complete germline development, as both male and female of this genotype were fertile (Figures 4D and S8C). Taken together, these observations both *in vitro* and *in vivo* suggest that Esrrb can substitute for Nanog function in germ cells.

DISCUSSION

Although conditional knockout approaches have shown that the Oct4 and Sox2 are essential for PGC development (Campolo et al., 2013; Kehler et al., 2004), studies on other pluripotency TFs have proved challenging. Here, we use conditional knockout strategies, mouse chimeras, and the recently developed PGCLC system (Hayashi et al., 2011) to assess the function of the Nanog-Esrrb axis in PGCs. This study establishes a role for Nanog in regulating PGC numbers *in vivo*. However, as for ESCs (Chambers et al., 2007), Nanog is strictly dispensable for PGC function. We show that knockin of *Esrrb* to the *Nanog* locus can complement the Nanog defect and is sufficient to rescue PGC numbers *in vivo*. Our study also supports a recently proposed role for Nanog in the maintenance of PGCLCs from pluripotent stem cells *in vitro* (Murakami et al., 2016), but expands on these findings, establishing that Esrrb can substitute for Nanog function in this system. Therefore, in addition to ESC self-renewal and iPSC reprogramming, Esrrb can functionally substitute for Nanog in PGC development. This strengthens the hypothesis that aspects of the naive pluripotency network are re-established in PGCs (Leitch and Smith, 2013). It will therefore be interesting to see whether the recently reported mitotic bookmarking activity of Esrrb in ESCs is also conserved in PGCs (Festuccia et al., 2016).

Previous experiments have suggested that Nanog is required for PGC development (Chambers et al., 2007; Yamaguchi et al., 2009). Although chimera experiments showed that *Nanog*$^{-/-}$ ESCs could form nascent PGCs at E11.5, *Nanog*-null PGCs

were not observed one day later at E12.5 (Chambers et al., 2007). This loss of PGCs was shown to be due to *Nanog* mutation, since repair of *Nanog* by homologous recombination restored E12.5 PGCs (Chambers et al., 2007). These findings were largely supported by a study in which induced knockdown of Nanog led to PGC death (Yamaguchi et al., 2009). More recently, however, *Nanog*$^{-/-}$ iPSCs were reported to be capable of germline transmission based on expression of a GFP transgene in tissues of chimera-derived offspring (Carter et al., 2014). Our present findings provide unequivocal evidence that PGC development can be completed in the absence of Nanog by showing that two newly derived *Nanog*$^{-/-}$ ESC lines exhibit germline transmission, as judged by coat color and the presence of *Nanog*-null alleles in F1 pups. Together with the severe reduction in PGC numbers observed in our conditional deletion experiments, this clarifies that the absence of NANOG compromises the development of the PGC population, but that individual PGCs can acquire full functionality in the absence of NANOG. This germline phenotype may render *Nanog*$^{-/-}$ PGCs disadvantaged compared with wild-type PGCs in the context of chimeras and reduce the frequency with which germline competency is observed. Our previous *Nanog*-null chimera experiments were performed using ESCs cultured in LIF/fetal calf serum (FCS). In contrast, both current examples of germline transmission were obtained using cells cultured in 2i/LIF, which may have enhanced the degree of chimerism, thereby increasing the likelihood of observing germline transmission, as previously shown for 3i/LIF culture medium (Kiyonari et al., 2010).

The fact that Esrrb can substitute for Nanog provides functional evidence that the naive pluripotency network may be conserved in PGCs. It is of interest that Esrrb fully restored PGC numbers by E12.5 when expressed from *Nanog*. Tetraploid embryos complemented by morula aggregation with *Esrrb*-null ESCs showed a reduction in PGC numbers of 50%–80% between E13.5 and E15.5 (Mitsunaga et al., 2004). Esrrb transcripts were first detected by real-time (RT)-PCR at E11.5 (Mitsunaga et al., 2004), with Nanog expression detected earlier in PGCs (Yamaguchi et al., 2005). However, re-analysis of published single-cell RNA sequencing (RNA-seq) data from PGCs (Hackett et al., 2013; Magnúsdóttir et al., 2013) shows that Esrrb and Nanog mRNAs both increase in expression from E6.5 to E7.5, remaining relatively steady thereafter until E12.5 (Figure S9). This suggests that ESRRB may function in PGCs before E11.5. Esrrb is also expressed during PGCLC differentiation, but at a reduced level in *Nanog*$^{-/-}$ PGCLCs relative to wild-type cells. The wild-type expression level of Esrrb mRNA is restored in *Nanog*$^{-/-}$ PGCLCs by Nanog induction. These results indicate that NANOG controls *Esrrb* expression in PGCs, but that, as is the case in ESCs, positive inputs in addition to NANOG also contribute to *Esrrb* expression (Festuccia et al., 2012; Martello et al., 2012).

Nanog$^{-/-}$ cells undergoing PGCLC differentiation showed increased apoptosis and reduced proliferation, validating previous important observations using an *in vivo* conditional knockdown approach (Yamaguchi et al., 2009). Consistent with this seminal study, apoptotic cells positive for active caspase-3 were invariably either OCT4 low or OCT4 negative. Restoring either Nanog or Esrrb expression in *Nanog*$^{-/-}$ PGCLCs is sufficient to reverse both the apoptosis and proliferation defects. Together, these studies add to the evidence that Esrrb is a physiologically relevant mediator of PGCLC function (Mitsunaga et al., 2004).

A limited number of studies have focused on other naive pluripotency factors in the germline. In addition to Oct4 and Sox2, conditional knockout of *Sall4* in PGCs does appear to affect gonadal PGC numbers, although interpretation is complicated by the mosaic deletion brought about by TNAP-Cre (Yamaguchi et al., 2015). The extent to which other pluripotency factors influence germline competence, PGC specification, and subsequent development is of significant interest. The PGCLC system may be an ideal tool to assess these factors. Recently, it was reported that induced expression of Nanog is sufficient to induce PGCLCs from epiblast-like cells (EpiLCs) (Murakami et al., 2016). Together with our data, this may indicate that Nanog has a dose-dependent influence on both the specification and maintenance of PGCs. This is reminiscent of the role of Nanog in ESCs, in which Nanog is not absolutely required, but functions as a pluripotency rheostat (Chambers et al., 2007; Mullin et al., 2017; 2008). In this regard, it is notable that *Prdm14* is a direct Nanog responsive gene in ESCs (Festuccia et al., 2012; 2013) and responds to Nanog in EpiLCs (Murakami et al., 2016). The ability of Esrrb to restore function *in vitro* to *Nanog*$^{-/-}$ PGCLCs further underscores the similarities between naive pluripotency and germline development. It would be interesting to assess whether elevated levels of Nanog or Esrrb *in vivo* might enhance PGC specification and germ cell numbers. How such manipulations of the pluripotency gene regulatory network might affect PGC identity is also of interest. This will enable us to reveal how the pluripotency gene regulatory network interacts with germ-cell-specific genes during PGC development and so build on the remarkably insightful studies that first pioneered the connection between pluripotency and the germline more than half a century ago (Stevens, 1983).

EXPERIMENTAL PROCEDURES

Animal studies were authorized by a UK Home Office Project License and carried out in a Home-Office-designated facility.

PGCLC Differentiation

PGCLC differentiation was performed essentially as described previously (Hayashi et al., 2011). Briefly, ESCs were cultured in 2i/LIF medium (as above) for several passages. Cells were then seeded onto fibronectin-coated plates at 1×10^5 cells/12 well in N2B27/1%KSR/bFGF/Activin A to obtain EpiLCs. Two days later, EpiLCs were collected and aggregated at 2,000 cells/well in PGCLC medium (50 ng/mL bone morphogenetic protein (BMP)4, 50 ng/mL BMP8a, 10 ng/mL stem cell factor [SCF], 10 ng/mL epidermal growth factor [EGF], and 1,000 U/mL LIF) using U-bottom 96-well plates (Thermo Fisher Scientific, 174925). For induction of gene expression, 1 μg/mL Dox (Sigma, D9891) was added at day 2 of PGCLC differentiation.

Further methods can be found in Supplemental Experimental Procedures.

SUPPLEMENTAL INFORMATION

Supplemental Information includes Supplemental Experimental Procedures, nine figures, and two tables and can be found with this article online at https://doi.org/10.1016/j.celrep.2017.12.060.

ACKNOWLEDGMENTS

Research in I.C.'s lab is supported by the Biotechnology and Biological Sciences (BBSRC) and the Medical Research Councils (MRC) of the United Kingdom (grant numbers BB/L002736/1 and MR/L018497/1, respectively). M.A.S. is a Wellcome Investigator, and his lab is supported by a core grant to the Gurdon Institute from the Wellcome Trust and Cancer Research UK. A.S. is an MRC Professor; the Cambridge Stem Cell Institute receives core funding from the Wellcome Trust and the MRC. N.F. was supported by an MRC studentship and an MRC Centenary award. H.G.L. was supported by a Merck, Sharp and Dohme Award from the University of Cambridge School of Clinical Medicine M.B./Ph.D. program and the James Baird Fund; he is now supported by the BBSRC, the MRC, and the National Institute for Health Research (NIHR) Imperial Biomedical Research Centre.

AUTHOR CONTRIBUTIONS

I.C., H.G.L., N.F., A.S., and M.A.S. conceived the project. H.G.L. performed the *Nanog* conditional knockout analyses. J.N. and H.G.L. derived the ESC lines. N.F. constructed the *Nanog*^{EsrrbKI} allele and performed initial analyses. M.Z. analyzed the *Nanog*^{EsrrbKI} phenotype and performed PGCLC experiments. W.W.C.T. and E.H.P. provided technical support. H.G.L., M.Z., and I.C. wrote the paper with input from all authors.

DECLARATION OF INTERESTS

The authors declare no competing interests.

Received: April 26, 2017
Revised: November 15, 2017
Accepted: December 17, 2017
Published: January 9, 2018

REFERENCES

Beddington, R.S. (1982). An autoradiographic analysis of tissue potency in different regions of the embryonic ectoderm during gastrulation in the mouse. J. Embryol. Exp. Morphol. *69*, 265–285.

Boroviak, T., Loos, R., Bertone, P., Smith, A., and Nichols, J. (2014). The ability of inner-cell-mass cells to self-renew as embryonic stem cells is acquired following epiblast specification. Nat. Cell Biol. *16*, 516–528.

Brook, F.A., and Gardner, R.L. (1997). The origin and efficient derivation of embryonic stem cells in the mouse. Proc. Natl. Acad. Sci. USA *94*, 5709–5712.

Campolo, F., Gori, M., Favaro, R., Nicolis, S., Pellegrini, M., Botti, F., Rossi, P., Jannini, E.A., and Dolci, S. (2013). Essential role of Sox2 for the establishment and maintenance of the germ cell line. Stem Cells *31*, 1408–1421.

Carter, A.C., Davis-Dusenbery, B.N., Koszka, K., Ichida, J.K., and Eggan, K. (2014). Nanog-independent reprogramming to iPSCs with canonical factors. Stem Cell Reports *2*, 119–126.

Chambers, I., Silva, J., Colby, D., Nichols, J., Nijmeijer, B., Robertson, M., Vrana, J., Jones, K., Grotewold, L., and Smith, A. (2007). Nanog safeguards pluripotency and mediates germline development. Nature *450*, 1230–1234.

Festuccia, N., Osorno, R., Halbritter, F., Karwacki-Neisius, V., Navarro, P., Colby, D., Wong, F., Yates, A., Tomlinson, S.R., and Chambers, I. (2012). Esrrb is a direct Nanog target gene that can substitute for Nanog function in pluripotent cells. Cell Stem Cell *11*, 477–490.

Festuccia, N., Osorno, R., Wilson, V., and Chambers, I. (2013). The role of pluripotency gene regulatory network components in mediating transitions between pluripotent cell states. Curr. Opin. Genet. Dev. *23*, 504–511.

Festuccia, N., Dubois, A., Vandormael-Pournin, S., Gallego Tejeda, E., Mouren, A., Bessonnard, S., Mueller, F., Proux, C., Cohen-Tannoudji, M., and Navarro, P. (2016). Mitotic binding of Esrrb marks key regulatory regions of the pluripotency network. Nat. Cell Biol. *18*, 1139–1148.

Hackett, J.A., Sengupta, R., Zylicz, J.J., Murakami, K., Lee, C., Down, T.A., and Surani, M.A. (2013). Germline DNA demethylation dynamics and imprint erasure through 5-hydroxymethylcytosine. Science *339*, 448–452.

Hayashi, K., Ohta, H., Kurimoto, K., Aramaki, S., and Saitou, M. (2011). Reconstitution of the mouse germ cell specification pathway in culture by pluripotent stem cells. Cell *146*, 519–532.

Ivanova, N., Dobrin, R., Lu, R., Kotenko, I., Levorse, J., DeCoste, C., Schafer, X., Lun, Y., and Lemischka, I.R. (2006). Dissecting self-renewal in stem cells with RNA interference. Nature *442*, 533–538.

Kehler, J., Tolkunova, E., Koschorz, B., Pesce, M., Gentile, L., Boiani, M., Lomelí, H., Nagy, A., McLaughlin, K.J., Schöler, H.R., and Tomilin, A. (2004). Oct4 is required for primordial germ cell survival. EMBO Rep. *5*, 1078–1083.

Kim, S., Günesdogan, U., Zylicz, J.J., Hackett, J.A., Cougot, D., Bao, S., Lee, C., Dietmann, S., Allen, G.E., Sengupta, R., and Surani, M.A. (2014). PRMT5 protects genomic integrity during global DNA demethylation in primordial germ cells and preimplantation embryos. Mol. Cell *56*, 564–579.

Kiyonari, H., Kaneko, M., Abe, S., and Aizawa, S. (2010). Three inhibitors of FGF receptor, ERK, and GSK3 establishes germline-competent embryonic stem cells of C57BL/6N mouse strain with high efficiency and stability. Genesis *48*, 317–327.

Kurimoto, K., Yabuta, Y., Ohinata, Y., Shigeta, M., Yamanaka, K., and Saitou, M. (2008). Complex genome-wide transcription dynamics orchestrated by Blimp1 for the specification of the germ cell lineage in mice. Genes Dev. *22*, 1617–1635.

Leitch, H.G., and Smith, A. (2013). The mammalian germline as a pluripotency cycle. Development *140*, 2495–2501.

Leitch, H.G., Nichols, J., Humphreys, P., Mulas, C., Martello, G., Lee, C., Jones, K., Surani, M.A., and Smith, A. (2013). Rebuilding pluripotency from primordial germ cells. Stem Cell Reports *1*, 66–78.

Leitch, H.G., Okamura, D., Durcova-Hills, G., Stewart, C.L., Gardner, R.L., Matsui, Y., and Papaioannou, V.E. (2014). On the fate of primordial germ cells injected into early mouse embryos. Dev. Biol. *385*, 155–159.

Magnúsdóttir, E., Dietmann, S., Murakami, K., Günesdogan, U., Tang, F., Bao, S., Diamanti, E., Lao, K., Göttgens, B., and Azim Surani, M. (2013). A tripartite transcription factor network regulates primordial germ cell specification in mice. Nat. Cell Biol. *15*, 905–915.

Martello, G., Sugimoto, T., Diamanti, E., Joshi, A., Hannah, R., Ohtsuka, S., Göttgens, B., Niwa, H., and Smith, A. (2012). Esrrb is a pivotal target of the Gsk3/Tcf3 axis regulating embryonic stem cell self-renewal. Cell Stem Cell *11*, 491–504.

Matsui, Y., Zsebo, K., and Hogan, B.L. (1992). Derivation of pluripotential embryonic stem cells from murine primordial germ cells in culture. Cell *70*, 841–847.

Mitsui, K., Tokuzawa, Y., Itoh, H., Segawa, K., Murakami, M., Takahashi, K., Maruyama, M., Maeda, M., and Yamanaka, S. (2003). The homeoprotein Nanog is required for maintenance of pluripotency in mouse epiblast and ES cells. Cell *113*, 631–642.

Mitsunaga, K., Araki, K., Mizusaki, H., Morohashi, K., Haruna, K., Nakagata, N., Giguère, V., Yamamura, K., and Abe, K. (2004). Loss of PGC-specific expression of the orphan nuclear receptor ERR-beta results in reduction of germ cell number in mouse embryos. Mech. Dev. *121*, 237–246.

Mullin, N.P., Yates, A., Rowe, A.J., Nijmeijer, B., Colby, D., Barlow, P.N., Walkinshaw, M.D., and Chambers, I. (2008). The pluripotency rheostat Nanog functions as a dimer. Biochem. J. *411*, 227–231.

Mullin, N.P., Gagliardi, A., Khoa, L.T.P., Colby, D., Hall-Ponsele, E., Rowe, A.J., and Chambers, I. (2017). Distinct contributions of tryptophan residues within the dimerization domain to Nanog function. J. Mol. Biol. *429*, 1544–1553.

Murakami, K., Günesdogan, U., Zylicz, J.J., Tang, W.W.C., Sengupta, R., Kobayashi, T., Kim, S., Butler, R., Dietmann, S., and Surani, M.A. (2016). NANOG alone induces germ cells in primed epiblast in vitro by activation of enhancers. Nature *529*, 403–407.

Ohinata, Y., Payer, B., O'Carroll, D., Ancelin, K., Ono, Y., Sano, M., Barton, S.C., Obukhanych, T., Nussenzweig, M., Tarakhovsky, A., et al. (2005). Blimp1 is a critical determinant of the germ cell lineage in mice. Nature *436*, 207–213.

Osorno, R., Tsakiridis, A., Wong, F., Cambray, N., Economou, C., Wilkie, R., Blin, G., Scotting, P.J., Chambers, I., and Wilson, V. (2012). The developmental dismantling of pluripotency is reversed by ectopic Oct4 expression. Development *139*, 2288–2298.

Resnick, J.L., Bixler, L.S., Cheng, L., and Donovan, P.J. (1992). Long-term proliferation of mouse primordial germ cells in culture. Nature *359*, 550–551.

Silva, J., Nichols, J., Theunissen, T.W., Guo, G., van Oosten, A.L., Barrandon, O., Wray, J., Yamanaka, S., Chambers, I., and Smith, A. (2009). Nanog is the gateway to the pluripotent ground state. Cell *138*, 722–737.

Smith, A. (2017). Formative pluripotency: the executive phase in a developmental continuum. Development *144*, 365–373.

Stevens, L.C. (1983). The origin and development of testicular, ovarian, and embryo-derived teratomas. In Cold Spring Harbor Conferences on Cell Proliferation, L.M. Silver, G.R. Martin, and S. Strickland, eds. (Cold Spring Harbor Laboratory Press), pp. 23–36.

Tam, P.P., and Zhou, S.X. (1996). The allocation of epiblast cells to ectodermal and germ-line lineages is influenced by the position of the cells in the gastrulating mouse embryo. Dev. Biol. *178*, 124–132.

Yamaguchi, S., Kimura, H., Tada, M., Nakatsuji, N., and Tada, T. (2005). Nanog expression in mouse germ cell development. Gene Expr. Patterns *5*, 639–646.

Yamaguchi, S., Kurimoto, K., Yabuta, Y., Sasaki, H., Nakatsuji, N., Saitou, M., and Tada, T. (2009). Conditional knockdown of Nanog induces apoptotic cell death in mouse migrating primordial germ cells. Development *136*, 4011–4020.

Yamaguchi, Y.L., Tanaka, S.S., Kumagai, M., Fujimoto, Y., Terabayashi, T., Matsui, Y., and Nishinakamura, R. (2015). Sall4 is essential for mouse primordial germ cell specification by suppressing somatic cell program genes. Stem Cell *33*, 289–300.

Cell Reports, Volume *22*

Supplemental Information

Esrrb Complementation Rescues Development of *Nanog*-Null Germ Cells

Man Zhang, Harry G. Leitch, Walfred W.C. Tang, Nicola Festuccia, Elisa Hall-Ponsele, Jennifer Nichols, M. Azim Surani, Austin Smith, and Ian Chambers

Figure S1.

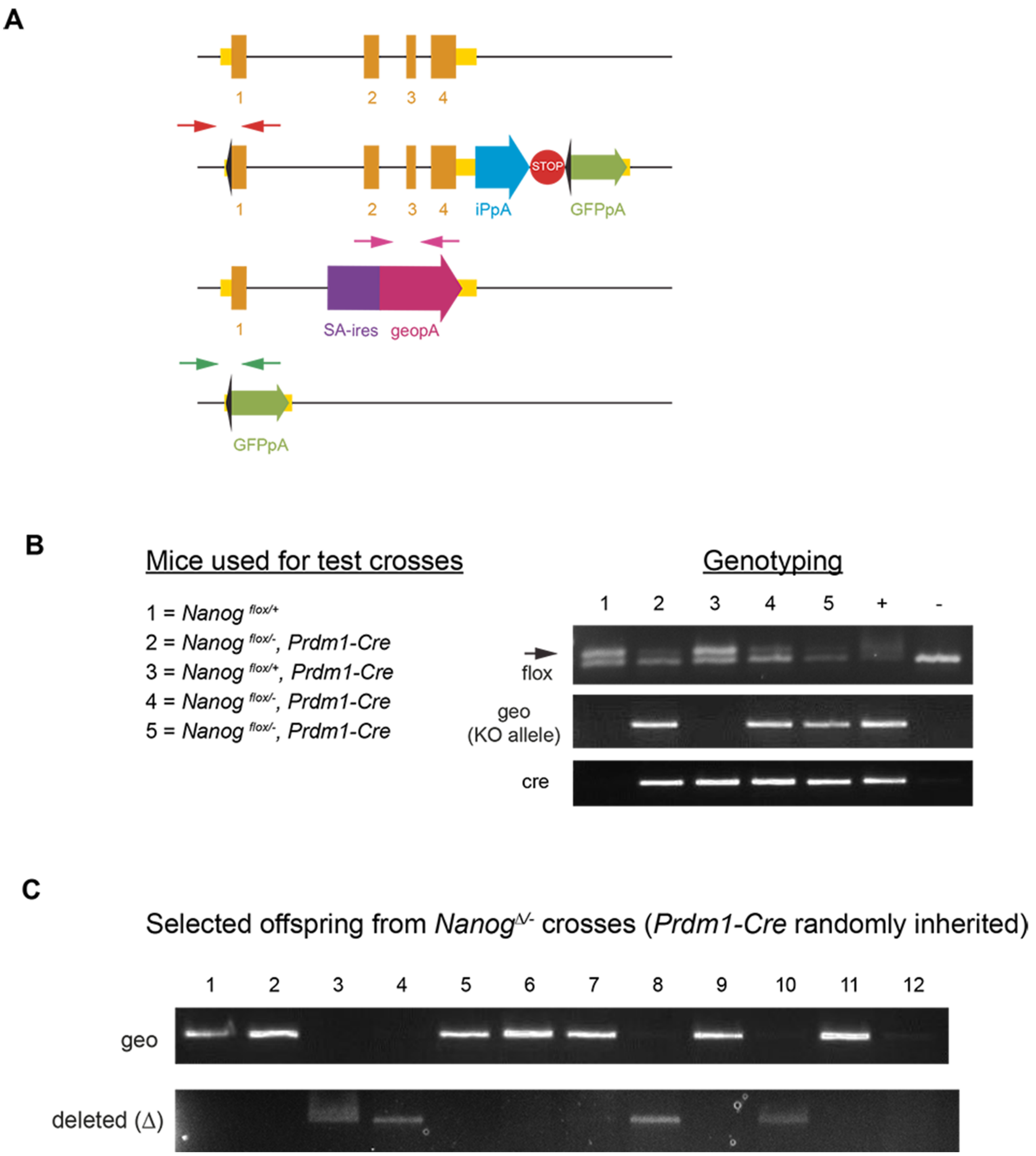

Figure S1. Genotyping of Nanog conditional deletion mice offspring. Related to Figure 1.
(A) Schematic of genotyping strategies.
(B) Genotyping of ear biopsies from adult *Nanog*$^{Δ/-}$ mice and control crosses.
(C) Genotyping of offspring derived from *Nanog*$^{Δ/-}$ x wildtype crosses. All offspring carry either the geo (null) or deleted allele.

Figure S2.

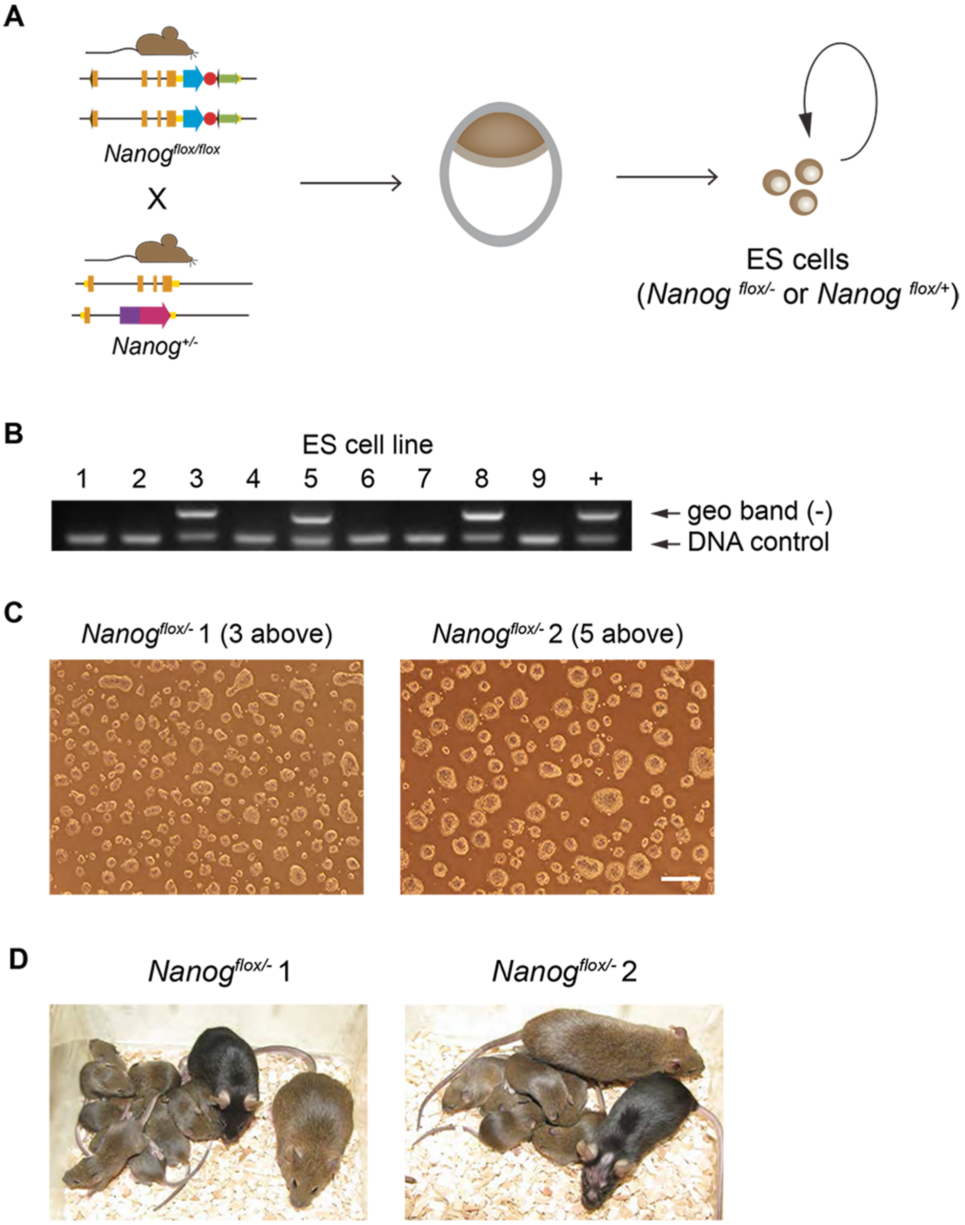

E

Line	Embryos	Chimaeras high	med	low	GLT
Nanog$^{flox/-}$ 1	32	15	0	2	1/3
Nanog$^{flox/-}$ 2	48	23	2	3	1/3

Figure S2. Derivation of germline competent *Nanog*$^{flox/-}$ ESCs. Related to Figure 2.
(A) Strategy for creating *Nanog*$^{flox/-}$ ESCs.
(B) Genotyping of ESCs derived from crosses as in A. Geo band indicates presence of null allele.
(C) Phase/contrast images of two *Nanog*$^{flox/-}$ ESC lines (bar = 100µm).
(D) High contribution coat colour chimaeras (generated by injection of agouti *Nanog*$^{flox/-}$ ESCs into C57BL/6 blastocysts), C57BL/6 mates, and agouti and black pups. Agouti pups indicate germline transmission of *Nanog*$^{flox/-}$ ESCs.
(E) Summary of blastocyst injections and germline transmission of *Nanog* $^{flox/-}$ ESCs.

Figure S3.

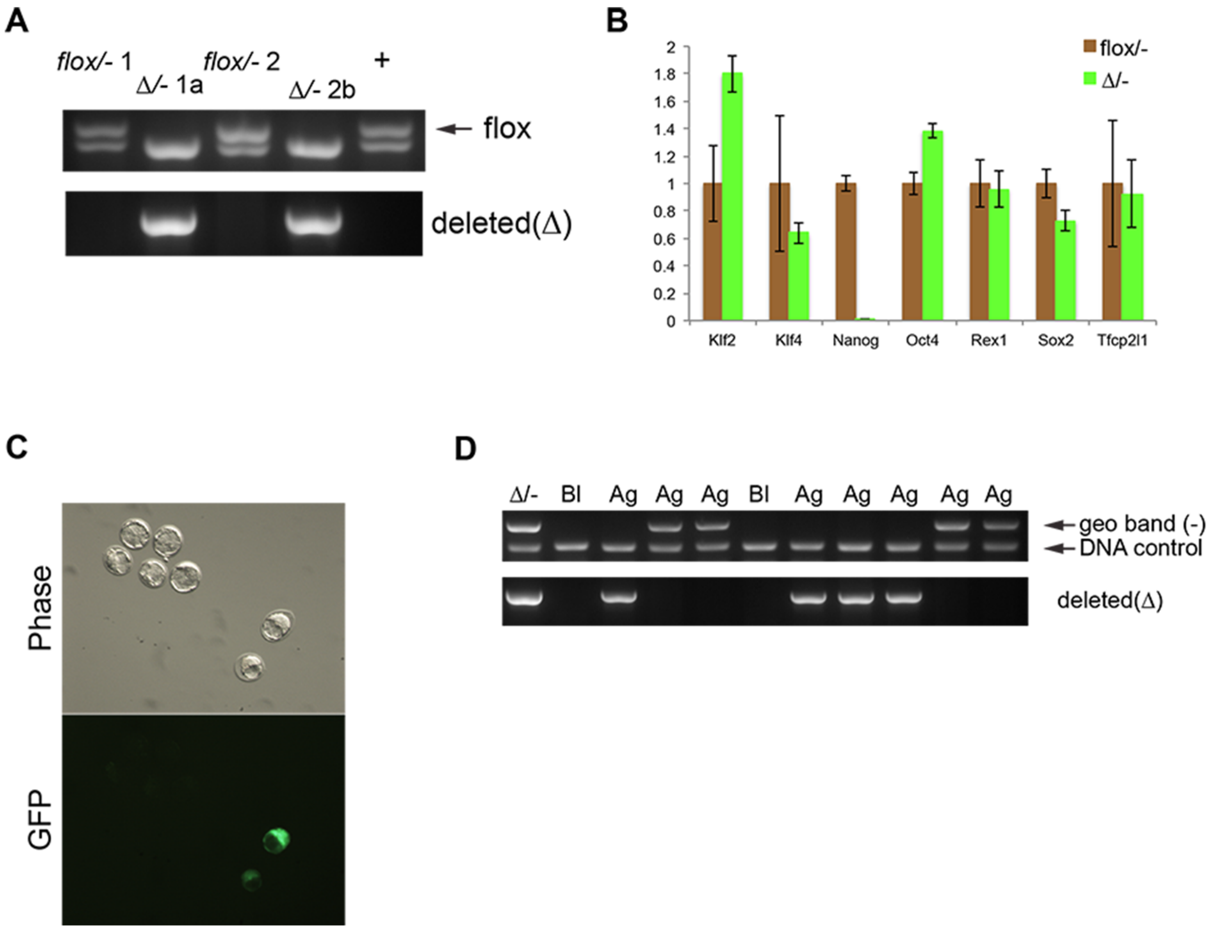

Figure S3. Further characterization of *Nanog*$^{\Delta/-}$ ESCs and their contribution to the germline. Related to Figure 2.
(A) Genotyping of clonal *Nanog*$^{\Delta/-}$ (*Nanog* null) ESC lines.
(B) Quantitative mRNA expression analysis of *Nanog* null and parental (*Nanog*$^{flox/-}$) ESCs. Each genotype is represented by two biological (1a, 1b, 2a, 2b) and two technical replicates. Error bars denote standard deviation.
(C) Phase and fluorescent images of blastocysts derived from chimaera x wildtype matings. Two blastocysts display GFP positive inner cell masses, indicating transmission of the *Nanog* deleted (Δ) allele.
(D) Genotyping of agouti (Ag) and black (Bl) offspring from chimaera x C57Bl/6 crosses. Bl offspring are positive for DNA control only. Ag offspring carry either the null (geo) or deleted (Δ) allele.

Figure S4.

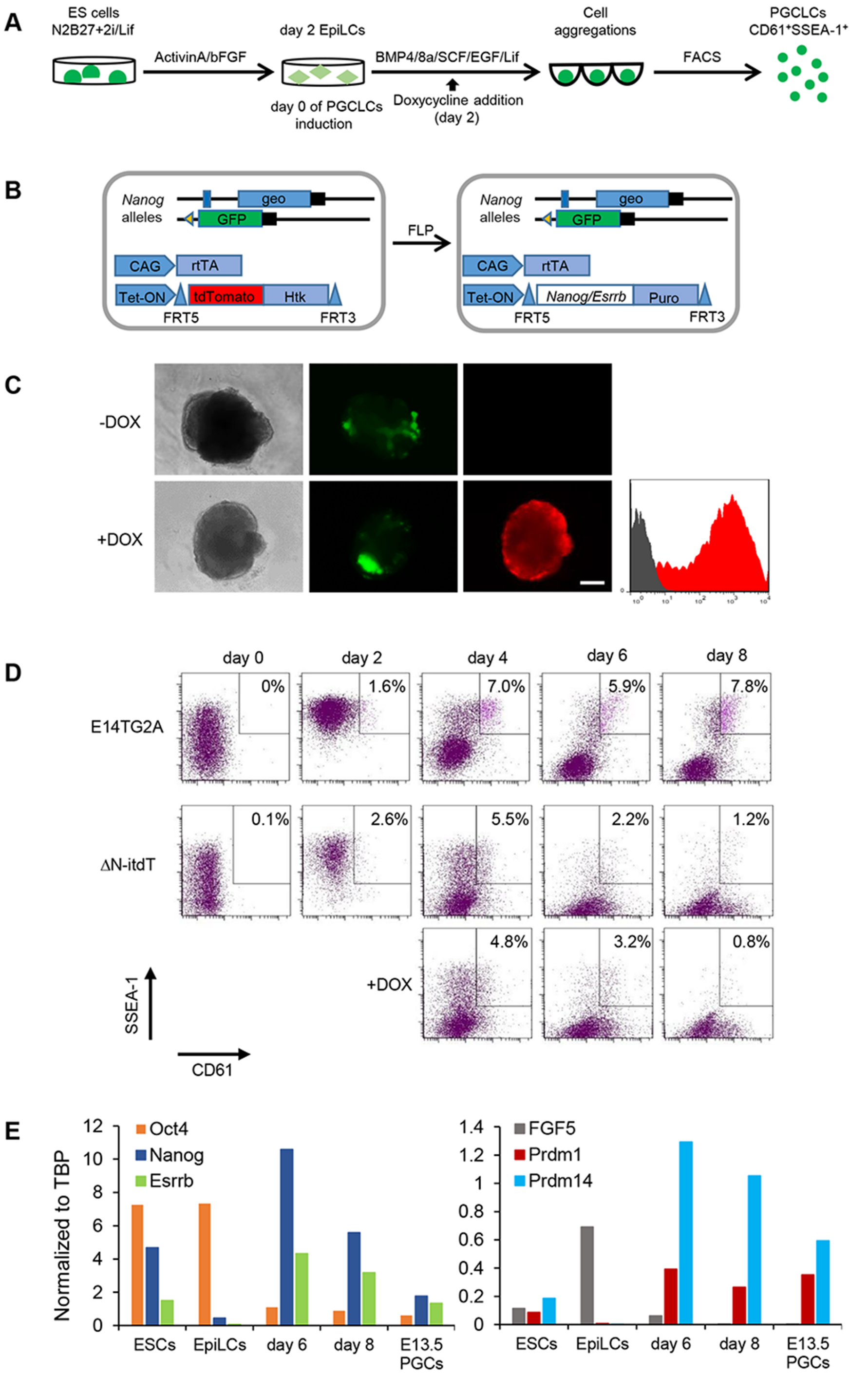

Figure S4. PGC differentiation in vitro. Related to Figure 3.
(A) Scheme for generating PGC like cells (PGCLCs) from ESCs.
(B) The strategy for generating the doxycycline inducible Nanog and Esrrb cell lines from the parental Doxycycline inducible tdTomato (itdT) Nanog null ESC line.
(C) ΔN-itdT cell aggregations, showing morphology, Nanog:GFP expression and FACS analysis of the tdTomato (grey, minus dox; red, plus dox) at in vitro PGC differentiation day8. To induce tdTomato, doxycycline was added at day 2 of PGCLC differentiation; bar = 200μm.
(D) FACS analysis for SSEA-1 and CD61 during E14TG2A and ΔN-itdT PGC differentiation. The percentage of $SSEA1^{+}/CD61^{+}$ cells are indicated.
(E) Quantitative mRNA analysis at the indicated days of PGCLC differentiation (day6 and 8 samples were first sorted for SSEA1 and CD61 expression). E13.5 genital ridges provide a control.

Figure S5.

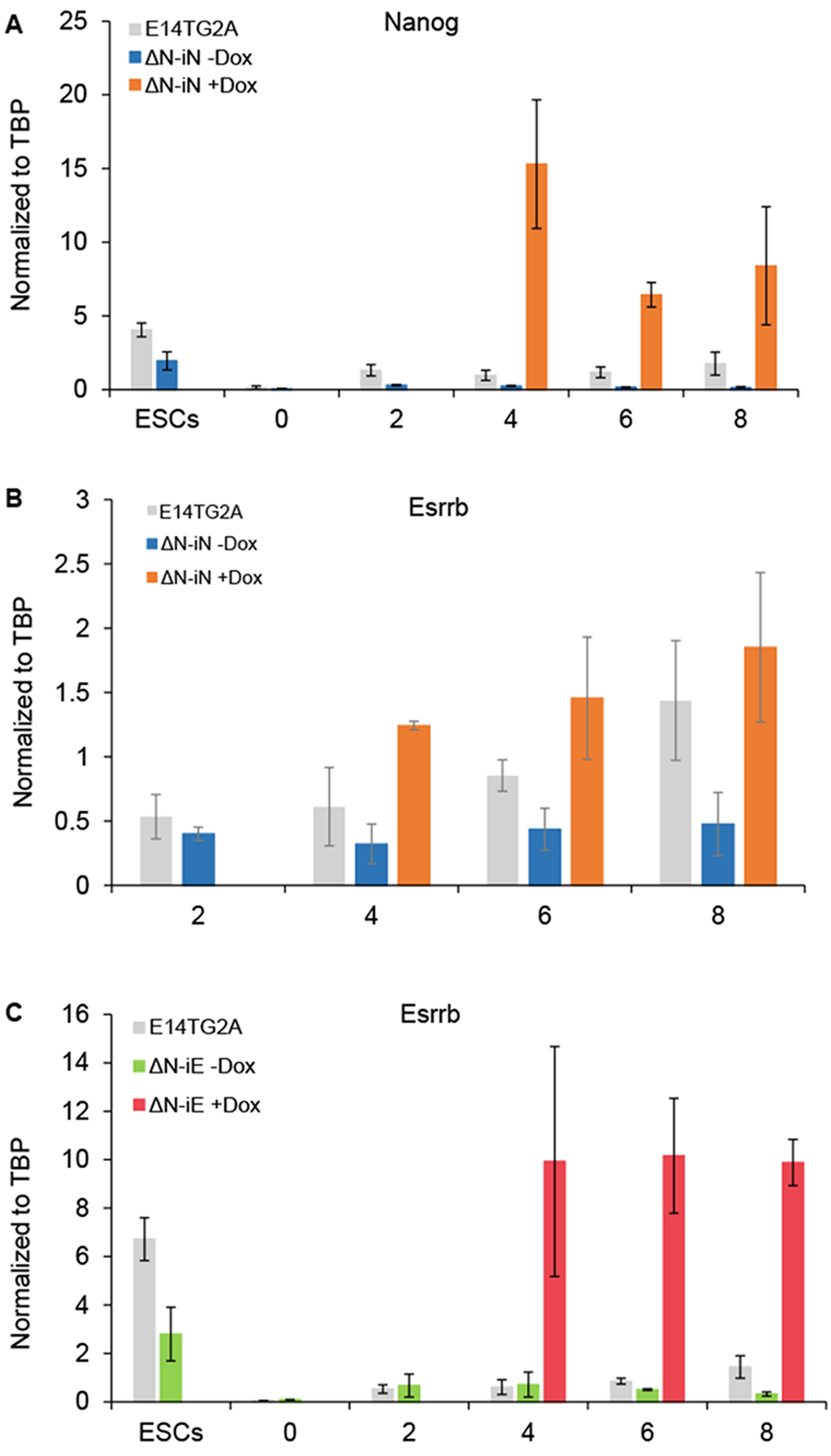

Figure S5. Quantitative transcript analysis during PGCLC differentiation of Nanog-/- derivative lines. Related to Figure 3

(A) Quantitative analysis of Nanog mRNA expression during PGCLC differentiation of wildtype and ΔN-iN cells in the presence or absence of Doxycycline at the indicated number of days. Values are means ± SD; n=3.

(B) Quantitative analysis of Esrrb mRNA expression during PGCLC differentiation of wildtype and ΔN-iN cells in the presence or absence of Doxycycline at the indicated number of days. Values are means ± SD; n=3.

(C) Quantitative analysis of Esrrb mRNA expression during PGCLC differentiation of wildtype and ΔN-iE cells in the presence or absence of Doxycycline at the indicated number of days. Values are means ± SD; n=3.

Figure S6.

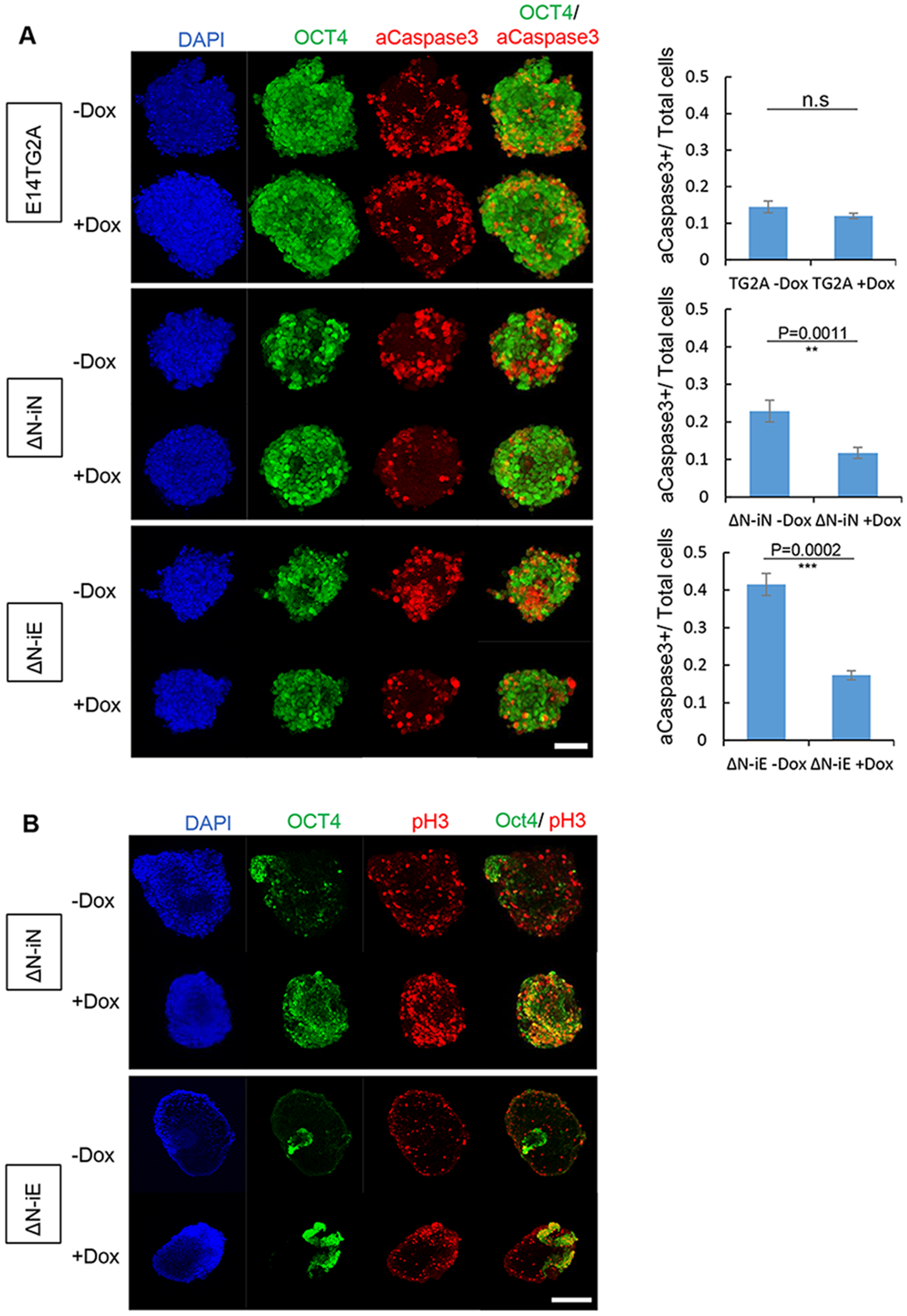

Figure S6. Apoptosis and cell proliferation in PGCLCs. Related to Figure 3.

(A) Confocal images of aggregated SSEA-1^{+}/$CD61^{+}$ cells analysed for OCT4 and active Caspase3 (aCaspase3) (left). Cells were sorted at day 6 of PGCLC differentiation, with Dox added at day 2 as indicated, and cultured for another 2 days. Images are maximum Z-stack projections. Bar=100µm. Quantitition of the proportion of cells positive for active Caspase3 in the population (right): values are means ± SD; n=3; p-values are indicated (unpaired t-test), "n.s", not significant.
(B) Confocal images of day 8 PGCLC aggregations analysed for OCT4 and phospho-Histone 3 (pH3), shown as maximum Z-stack projections. Bar= 200µm.

Figure S7.

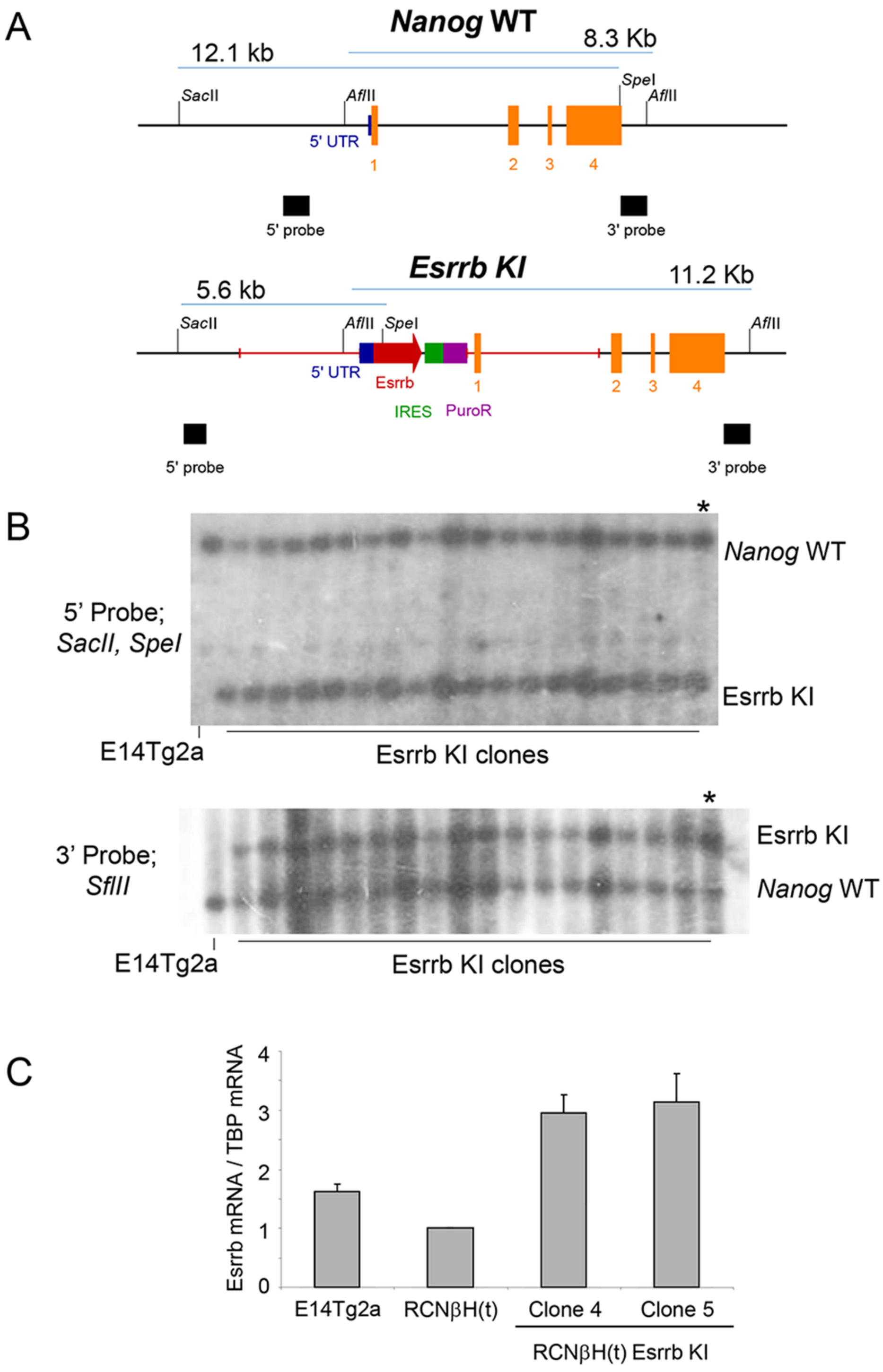

Figure S7. Derivation of E14Tg2a *Nanog*^{EsrrbKI} ESC lines. Related to Figure 4

(A) Schematic representation of the structure of the *Nanog* locus in E14Tg2a and *Nanog*^{EsrrbKI} derivative ESC lines, showing wild-type and targeted *Nanog* alleles, along with restriction sites and DNA probes used for Southern analysis. The expected sizes of the DNA fragments obtained after digestion are shown on top of each diagram. The homology arms of the targeting vector are shown in red.

(B) Southern blots performed on DNA samples from E14Tg2a and puromycin-resistant subclones. *: clone 18 was used to derive mice by blastocyst injection.

(C) Quantitative Esrrb transcript analysis in wild-type, *Nanog*-null and two lines in which one of the *Nanog*-null alleles in RCNβH(t) was rescued by knock-in of the Esrrb targeting vector used in (A).

Figure S8.

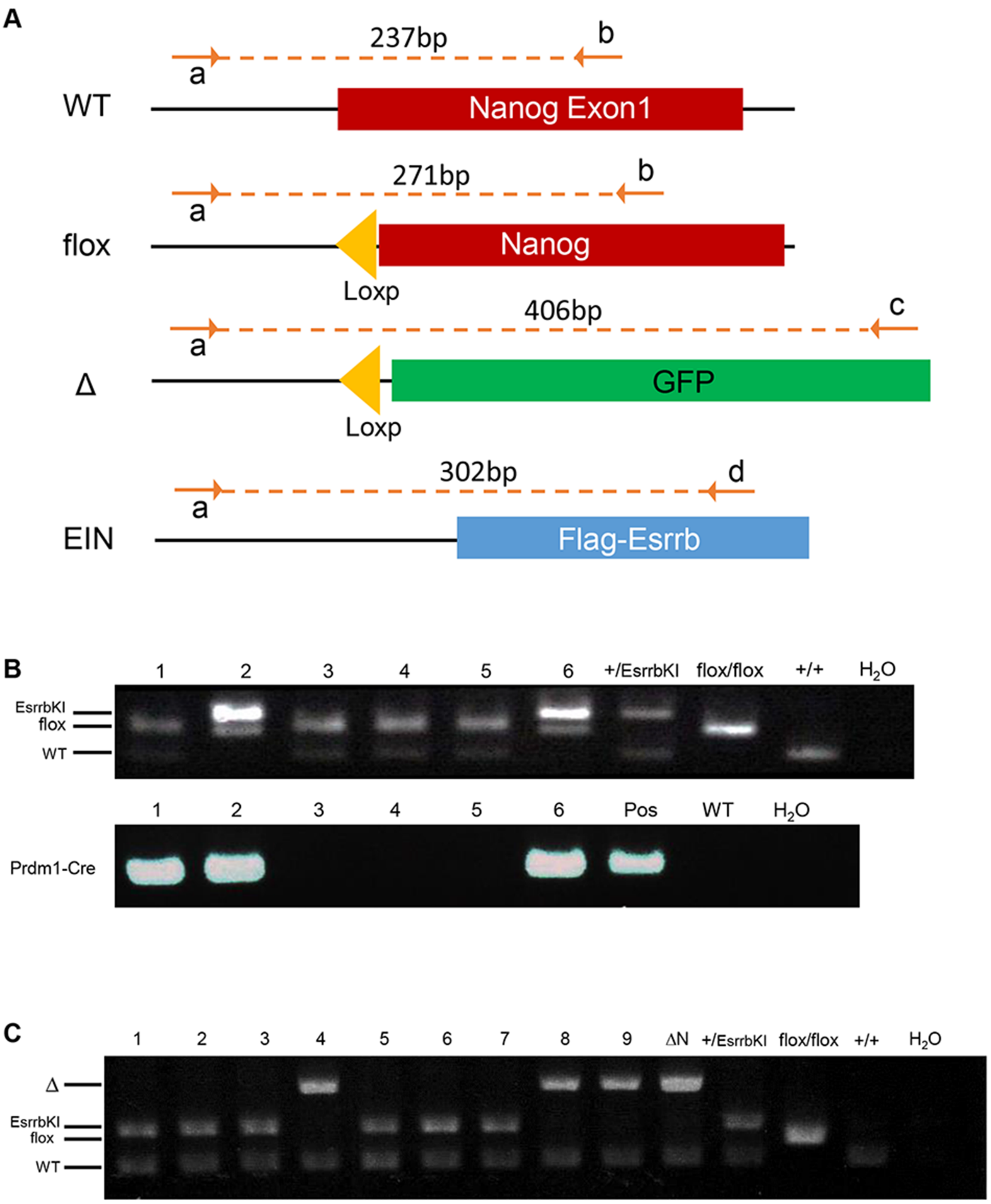

Figure S8. Genotyping of embryos and offspring from crosses of *Nanog*$^{+/EsrrbKI}$ mice. Related to Figure 4.

(A) The genotyping strategies to identify *Nanog* alleles showing wild-type, loxP-flanked alleles before and after excision and Esrrb knock-in.
(B) E12.5 embryos generated by crossing *Nanog*$^{flox/flox}$ female mice with *Prdm1-Cre*: *Nanog*$^{+/EsrrbKI}$ male mice were assessed by PCR. *Nanog*$^{+/EsrrbKI}$, *Nanog*$^{flox/flox}$, *Nanog*$^{+/+}$ DNA samples or H_2O (lanes 7-10) were used as controls.
(C) Genotyping of litters from crosses between adult *Nanog*$^{\Delta/EsrrbKI}$ and wild-type mice. *Nanog*$^{+/\Delta}$ ESCs (ΔN), *Nanog*$^{+/EsrrbKI}$, *Nanog*$^{flox/flox}$, *Nanog*$^{+/+}$ DNA samples or H_2O (lanes 10-14) were used as controls.

Figure S9.

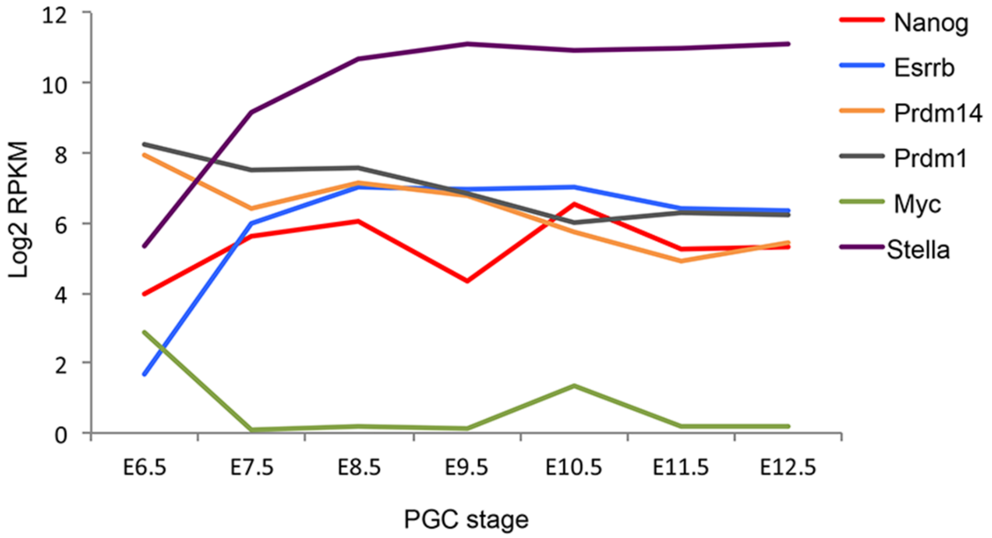

Figure S9. *Esrrb* expression in primordial germ cells. Related to Figures 3 and 4.
Single-cell RNA-seq analysis of Nanog, Esrrb, Prdm1, Prdm15 and Myc expression. Shown as $\log_2$ reads per kilobase of transcript per million mapped reads (RPKM). Primary data from Hackett et al., 2013.

Table S1. All oligonucleotide sequences are given 5' - 3'. Related to Figure 3.

Primers for qPCR	
Primer name	Sequence
Blimp1 Frw	ttctcttggaaaaacgtgtggg
Blimp1 Rev	ggagccggagctagacttg
Prdm14 Frw	tcaattcactcccgaagtacca
Prdm14 Rev	ccggggatggcagaagtaaa
Fgf5 Frw	tgtgtctcaggggattgtagg
Fgf5 Rev	agctgttttcttggaatctctcc
Nanog Frw	tccccacagtttgcctagtt
Nanog Rev	ttctcgggatgaaaaactgc
Oct4 Frw	ttccaccaggcccccc
Oct4 Rev	ggtgagaaggcgaagtctgaag
Esrrb Frw	cgattcatgaaatgcctcaa
Esrrb Rev	cctcctcgaactcggtca
TBP Frw	ggggagctgtgatgtgaagt
TBP Rev	ccaggaaataattctggctca

Table S2. All oligonucleotide sequences are given 5’ -3’.

Primers for Genotyping			
**Primer name**	**Sequence**	**Target(s)**	**Size (bp)**
Prdm1Cre Frw	gccgaggtgcgcgtcagtac	Cre	215
Prdm1Cre Rev	ctgaacatgtccatcaggttcttg		
Nanog locus Frw	gctgcggctcacttccttctgact	WT & flox	237 & 271
Nanog locus Rev1	aggcattgatgaggcgttcccagaatt		
Nanog locus Rev2	gctggatggctccgtcttgatgaa	EsrrbKI	302
Nanog locus Rev3	ggacttgaagaagtcgtgctg	Δ	
Nanog locus Fwd 2	gggtcaccttacagcttcttttgcatta	Deleted	
Nanog locus Rev4	gacttgaagaagtcgtgctgcttcatg		
Bgeo Frw	gttgcagtgcacggcagatacacttgctga	βgeo	
Bgeo Rev	gccactggtgtgggccataattcaattcgc		
TCRD Fwd	caaatgttgcttgtctggtg	DNA (control)	
TCRD Rev	gtcagtcgagtgcacagttt		

Supplemental Experimental Procedures

ESC derivation

Nanog mutant ESC lines were derived as described previously (Nichols et al., 2009). Primary colonies were picked and expanded in 2i/LIF medium (Ying et al., 2008). 2i/LIF medium comprises N2B27 basal medium (Ying et al., 2003) supplemented with 1µM PD0325901, (Axon Medchem), 3 µM CHIR99021 (Axon Medchem) and mouse LIF (prepared in house). Single cell deposition was performed using a MoFlo high-speed cell sorter (Dako Cytomation).

Chimera Production

Chimaeras were produced by microinjection of ESCs (agouti) into C57Bl/6 blastocysts (Nagy et al., 2003).

Immunostaining

For immunostaining of ESCs, cells were fixed in 4% paraformaldehyde (PFA) (10 min, RT) then blocked and permeabilized in PBS/0.1% Triton X-100/1% BSA. Primary antibodies were incubated in the same buffer (overnight, 4C). Secondary antibodies were incubated for 1hr (RT). Cells were washed (3x, 15 min) in PBS after primary and secondary antibody incubations. Nuclei were stained with DAPI. Primary antibodies were: OCT4 (BD, 1:200), NANOG (EBiosciences 1:200) and GFP (abcam, 1:800). Nuclei were stained with DAPI. Alexa Fluor secondary antibodies (Invitrogen) were used at 1:500 dilution.

For wholemount immunostaining, dissected gonads (from E11.5 or E12.5) or PGCLC aggregations were washed twice in PBS containing 3mg/ml polyvinylpyrrolidone (PBS/PVP) , fixed in 4% PFA (20min, RT) and washed three times in PBS/PVP. Samples were then permeabilised in 0.3% Triton X-100 PBS/PVP (45min, RT), blocked (2hours, RT) in PBS / 0.1%BSA / 0.01% Tween20 / 3% goat serum (blocking buffer). Gonads were then incubated with 1:800 chicken anti-GFP antibody (abcam, ab13970), 1:200 rat anti-Nanog antibody (ebioscience, 14-5761-80), 1:100 goat anti-Dazl antibody (Santacruz, sc-27333), 1:500 rabbit anti-DAZL (Abcam ab34139) or 1:500 goat anti-DDX4 (R&D AF2030) diluted in blocking buffer (overnight, 4°C). Aggregations were incubated with either 1:400 rabbit anti-cleaved Caspase3 (Cell signalling, 9661) or 1:200 rabbit anti-phospho Histone 3 (Cell signalling, 53348) combined with 1:200 goat anti-Oct3/4 (Santacruz, sc-8628) diluted in blocking buffer (overnight, 4 °C). The following day, unbound primary antibody was removed by rinsing in 10% blocking buffer/PBS (3×, 15min) and then incubated with the appropriate secondary antibodies. These were 1:500 FITC donkey anti Chicken (abcam), 1:1000 Aldrich 568 Donkey Anti-Rat (sigma, SAB4600077), 1:1000 Alexa Fluor 568 Donkey Anti-rabbit (Invitrogen, A10042) and 1:500 Alexa Fluor 647 Donkey Anti-Goat (Invitrogen, A21447) (3hours, RT). After washing in 10% blocking buffer (3×, 15min), gonads were mounted on glass microscope slides. Aggregations were incubated with DAPI for nuclear staining (overnight, 4°C) and then treated sequentially for 5 mins in 10%, 25%, 50%, 97% thiodiethanol (sigma 166782) before imaging on a Leica SP8 STED-CW Confocal microscope.

FACS analysis

FACS was as described (Zhang et al., 2014). For E14TG2A, ΔN-iN and ΔN-iE cells, Alexa Fluor® 647 anti-CD15(SSEA-1) (Biolegend, 125608) and PE anti-CD61 (Biolegend, 104307) antibodies were used and DAPI was used to gate out dead cells. For ΔN-iTdt cells, Brilliant Violet 421™ anti-CD15 (SSEA-1) (Biolegend, 125613) and APC anti-CD61 (Biolegend, 104315) antibodies were used. The concentration of antibody was tested first. Isotype control antibody Alexa Fluor® 647 Mouse IgM (Biolegend, 401618) and PE Armenian Hamster IgG (Biolegend, 400908) were used to set the gates. Cells were sorted on a BD FACS Aria II and were analyzed on a BD LSR Fortessa (5 laser) analyser.

RNA analysis

Cells were isolated in RLT buffer (Qiagen, 74104) supplemented with 1% β-mercaptoethanol. Cell lysates were homogenized with QIAshredder columns (Qiagen, 79656). Total RNA, free of genomic DNA, was purified using RNeasy mini kit (Qiagen, 74104) and reverse transcribed using the SuperScript® III Reverse Transcriptase kit (Invitrogen, 18080093). Quantitative real-time PCR was done with LightCycler 480 SYBR Green I Master mix (Roche, 04887352001) and LightCycler 480 II machine (Roche, 05015243001). Primers are listed in Table S1. For Nanog null and control ESCs, TaqMan probes for Klf2, Klf4, Nanog, Oct4/Pou5f1, Rex1/Zfp42, Tfcp2l1, Sox2 and Gapdh were used.

Genotyping

Ear notches from juvenile mice, embryos biopsies or ESC pellets were incubated in lysis buffer (1x Qiagen PCR buffer (201205) supplemented with 0.5% Tween 20, 0.5% NP40 and 10µg/ml Proteinase K (Sigma, P2308)) at 56 °C for >3 hours. Proteinase K was then heat-inactivated (95 °C, 10 mins). After centrifugation, 5 µl of lysate was used for genotyping. PCRs were performed with Taq Polymerase (Qiagen 201205) in 30 µl final volume. Primers are listed in Table S2. PCR products were separated on 2% TBE agarose gels and visualised via Ethidium Bromide staining.

Supplemental References

Nichols, J., Silva, J., Roode, M., and Smith, A. (2009). Suppression of Erk signalling promotes ground state pluripotency in the mouse embryo. Development *136*, 3215–3222.

Ying, Q.-L., Stavridis, M., Griffiths, D., Li, M., and Smith, A. (2003). Conversion of embryonic stem cells into neuroectodermal precursors in adherent monoculture. Nat Biotech *21*, 183–186.

Ying, Q.-L., Wray, J., Nichols, J., Batlle-Morera, L., Doble, B., Woodgett, J., Cohen, P., and Smith, A. (2008). The ground state of embryonic stem cell self-renewal. Nature *453*, 519–523.

Zhang, M., Zhou, H., Zheng, C., Xiao, J., Zuo, E., Liu, W., Xie, D., Shi, Y., Wu, C., Wang, H., et al. (2014). The roles of testicular c-kit positive cells in de novo morphogenesis of testis. Sci. Rep. *4*, 5936.
